# Epiblast Stem Cell-Based System Reveals Reprogramming Synergy of Germline Factors

**DOI:** 10.1016/j.stem.2012.01.020

**Published:** 2012-04-06

**Authors:** Astrid Gillich, Siqin Bao, Nils Grabole, Katsuhiko Hayashi, Matthew W.B. Trotter, Vincent Pasque, Erna Magnúsdóttir, M. Azim Surani

**Affiliations:** 1 Wellcome Trust/Cancer Research UK Gurdon Institute, University of Cambridge, Tennis Court Road, Cambridge CB2 1QN, UK; 2 Department of Anatomy and Cell Biology, Graduate School of Medicine, Kyoto University, Yoshida-Konoe-cho, Sakyo-ku, Kyoto 606-8501, Japan; 3 Anne McLaren Laboratory for Regenerative Medicine, University of Cambridge, Robinson Way, Cambridge CB2 0SZ, UK

## Abstract

Epigenetic reprogramming in early germ cells is critical toward the establishment of totipotency, but investigations of the germline events are intractable. An objective cell culture-based system could provide mechanistic insight on how the key determinants of primordial germ cells (PGCs), including Prdm14, induce reprogramming in germ cells to an epigenetic ground state. Here we show a *Prdm14-Klf2* synergistic effect that can accelerate and enhance reversion of mouse epiblast stem cells (epiSCs) to a naive pluripotent state, including X reactivation and DNA demethylation. Notably, *Prdm14* alone has little effect on epiSC reversion, but it enhances the competence for reprogramming and potentially PGC specification. Reprogramming of epiSCs by the combinatorial effect of *Prdm14-Klf2* involves key epigenetic changes, which might have an analogous role in PGCs. Our study provides a paradigm toward a systematic analysis of how other key genes contribute to complex and dynamic events of reprogramming in the germline.

## Graphical abstract

**Graphical Abstract:**

**Figure:**
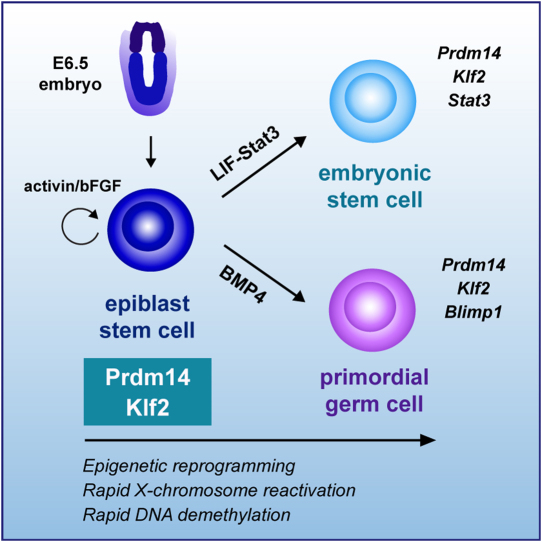

**Highlights:**

► *Prdm14* synergizes with *Klf2* to accelerate reversion of epiblast stem cells ► *Prdm14* has little effect on its own, but enhances competence for reprogramming ► Reversion by *Prdm14* and *Klf2* occurs efficiently in the absence of Blimp1 ► EpiSC reversion is useful to explore the role of germline factors in reprogramming

## Introduction

Specification of primordial germ cells (PGCs) in mice is accompanied by extensive epigenetic reprogramming, which is essential for generating the totipotent state (Hayashi and Surani, 2009a). The key determinants of PGC specification, Blimp1/Prdm1 and Prdm14, induce repression of the somatic program and initiate epigenetic reprogramming in early germ cells (Ohinata et al., 2005; Vincent et al., 2005; Yamaji et al., 2008), and they regulate this process together with their direct and indirect targets. Cell culture-based systems might be particularly useful for testing how the individual components contribute to complex reprogramming events in the germline, which in turn could improve our ability to control cell fates.

PGC specification commences at embryonic day (E) 6.25 from postimplantation epiblast; these epiblast cells undergo major epigenetic changes after implantation, including DNA methylation and X inactivation (Hayashi and Surani, 2009a). Epiblast stem cells (epiSCs) derived from E5.5–E6.5 epiblast inherit key properties from these cells (Brons et al., 2007; Tesar et al., 2007) and retain the potential to undergo reversion to embryonic stem cells (ESCs) (Bao et al., 2009) or specification to unipotent PGCs (Hayashi and Surani, 2009b). The alternative fates from epiSCs to ESCs or PGCs are quite distinct, but they share important common features, including reactivation of the inactive X chromosome, DNA demethylation, and re-expression of key pluripotency genes (Hayashi and Surani, 2009a). Importantly, for *Oct4* expression, there is a switch from the use of the proximal to the distal enhancer, the so-called enhanceosome locus of pluripotency (Bao et al., 2009; Chen et al., 2008; Yeom et al., 1996). Thus, the key epigenetic modifications in postimplantation epiblast and epiSCs, which constitutes a robust epigenetic boundary, are reversed during reprogramming in both instances, although reversion of epiSCs to ESCs, or indeed of somatic cells to induced pluripotent stem cells (iPSCs), may also transit through a PGC-like state (Chu et al., 2011). EpiSCs can therefore be used to investigate aspects of epigenetic reprogramming and the roles of genes in early germ cells. The fact that epiSCs acquire additional DNA methylation during their derivation, which probably reduces their competence for PGC specification (Bao et al., 2009; Hayashi and Surani, 2009b), is paradoxically an advantage for their use in such assays.

EpiSCs self-renew in activin and basic fibroblast growth factor (bFGF), with a gene expression profile and epigenetic state that is distinct from mouse ESCs (Brons et al., 2007; Tesar et al., 2007). EpiSCs can, however, revert to ESCs upon exposure to leukemia inhibitory factor (LIF)-Stat3 signaling on feeder cells (Bao et al., 2009; Yang et al., 2010), a process that is improved with the introduction of transcription factors, such as *Klf4* or *Nr5a2* (Guo and Smith, 2010; Guo et al., 2009).

Here we used epiSCs to explore the role of germline factors during reprogramming to ESCs. We found a potent combinatorial role for early germline factors, *Prdm14-Klf2*, that accelerate and enhance the ensuing process, including X reactivation and DNA demethylation, which are among the key reprogramming events that are also seen in early PGCs. Our approach might provide critical insight into the role of key germline factors, which in turn could be tested directly on germ cells while expanding our knowledge of complex reprogramming mechanisms in general.

## Results

### EpiSC-Based Assay for Reprogramming and X Reactivation

There are two major attributes of epiSCs that can be utilized to generate reporter lines for studying epigenetic reprogramming. First, female epiSCs exhibit an inactive X chromosome (Xi), which is unlike ESCs and PGCs (Guo et al., 2009; Hayashi and Surani, 2009b). Second, the expression of *Oct4* in epiSCs requires its proximal enhancer (PE), whereas it is the distal enhancer (DE) that drives *Oct4* expression in both ESCs and PGCs (Bao et al., 2009; Yeom et al., 1996). We therefore established two epiSC reporter lines to examine reprogramming by monitoring the status of X reactivation and by analyzing the activation of *Oct4* DE in response to germline factors (see also later).

**Figure 1:**
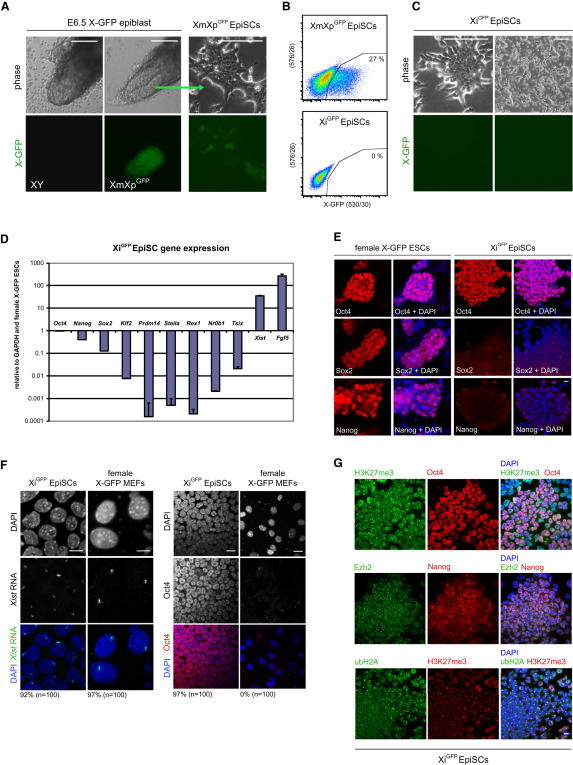
Establishment and Characterization of Xi^GFP^ EpiSCs (A) Phase contrast and fluorescence images of XmXp^GFP^ epiSCs derived from female E6.5 X-GFP epiblast. Scale bars represent 100 μm. (B) Flow cytometry analysis of XmXp^GFP^ epiSCs and Xi^GFP^ epiSCs. (C) Phase contrast and fluorescence images of Xi^GFP^ epiSCs cultured in activin and bFGF on fibronectin. Scale bars represent 100 μm. (D) Q-PCR analysis for selected ESC and epiSC markers in Xi^GFP^ epiSCs relative to GAPDH and ESCs. Error bars are mean ± SD (n = 2). (E) Immunostaining for Oct4, Sox2, and Nanog in Xi^GFP^ epiSCs and ESCs. Nuclei were stained with DAPI. Scale bar represents 10 μm. (F) RNA-FISH for *Xist* and side-by-side immunostaining for Oct4 in Xi^GFP^ epiSCs and female MEFs. Nuclei were stained with DAPI. Scale bars represent 10 μm (*Xist*), 20 μm (Oct4). (G) Double immunostaining for H3K27me3/Ezh2/ubH2A (green) and Oct4/Nanog/H3K27me3 (red) in Xi^GFP^ epiSCs. Nuclei were stained with DAPI. Scale bar represents 10 μm. See also Figure S1.

To monitor the state of the X chromosome in epiSCs, we derived epiSCs from female E6.5 epiblast with a GFP reporter on the paternal X chromosome (Hadjantonakis et al., 2001). The resulting XmXp^GFP^ epiSC lines showed heterogeneous GFP expression resulting from random X chromosome inactivation in female postimplantation epiblast (Figures 1A and 1B). Next, we established a homogeneous population of GFP-negative epiSCs, termed Xi^GFP^ epiSCs (Figure 1B), in which the GFP transgene is located exclusively on the Xi, providing a basis to study the reactivation of the Xi by monitoring GFP expression.

We observed a stable Xi when the cells were cultured in the absence of feeder cells on fibronectin in serum-free medium containing activin and bFGF, as shown by the fact that we did not detect GFP-positive cells (Figures 1B and 1C). Only when we cultured these Xi^GFP^ epiSCs on feeder cells in the presence of serum supplement did we notice, albeit very infrequently, a small number (around 0.05%) of GFP-positive cells (Pasque et al., 2011; data not shown), which may arise because of LIF or unknown factors secreted by feeder cells or present in serum supplement. We therefore cultured the cells without feeders on fibronectin and without serum supplement to ensure a repressed X-GFP reporter at the outset.

Xi^GFP^ epiSCs showed high expression of postimplantation epiblast genes *Fgf5* and *Xist* and low expression of ESC- and PGC-associated genes, including *Klf2*, *Prdm14*, *Stella*, *Rex1, Nr0B1*, and *Tsix* (Figure 1D; Guo et al., 2009). Whereas Oct4 transcript and protein levels were similar in Xi^GFP^ epiSCs and female ESCs, both Sox2 and Nanog were detected at reduced levels in Xi^GFP^ epiSCs (Figures 1D and 1E; Han et al., 2010). Furthermore, Oct4-positive Xi^GFP^ epiSCs showed nuclear domains of *Xist* (92%, n = 100), as was the case in female mouse embryonic fibroblasts (MEFs) (97%, n = 100) (Figure 1F), consistent with the presence of an Xi (Brockdorff et al., 1991). These Xi^GFP^ epiSCs showed accumulation of the repressive histone 3 lysine 27 trimethylation (H3K27me3) chromatin mark together with nuclear foci of Enhancer of Zeste (Ezh2) (Figure 1G; Pasque et al., 2011). In addition, monoubiquitinated H2A (ubH2A) (de Napoles et al., 2004) colocalized with H3K27me3 domains in the majority of epiSC nuclei (Figure 1G). Notably, we did not detect binding of Oct4, Sox2, or Nanog to intron 1 of *Xist* in Xi^GFP^ epiSCs, unlike female ESCs (Figure S1A available online; Navarro et al., 2008), which indicates dissociation of key pluripotency factors from *Xist* intron 1 (and perhaps other loci), probably during the formation of postimplantation epiblast.

Thus, the presence of multiple Xi markers demonstrates stable X inactivation in Xi^GFP^ epiSCs under our culture conditions.

### Germline Factors with Impact on Reprogramming and X Reactivation

Next, we asked whether reprogramming and X chromosome reactivation in epiSCs could be promoted by factors that are upregulated in early germ cells, including *Blimp1, Prdm14, Stella, Tcfap2c, Klf2, Klf5, Nanog, Sox2*, and *Dnd1*, because these genes are induced just before the repression of *Xist* and X reactivation in PGCs (Chuva de Sousa Lopes et al., 2008; Kurimoto et al., 2008).

**Figure 2:**
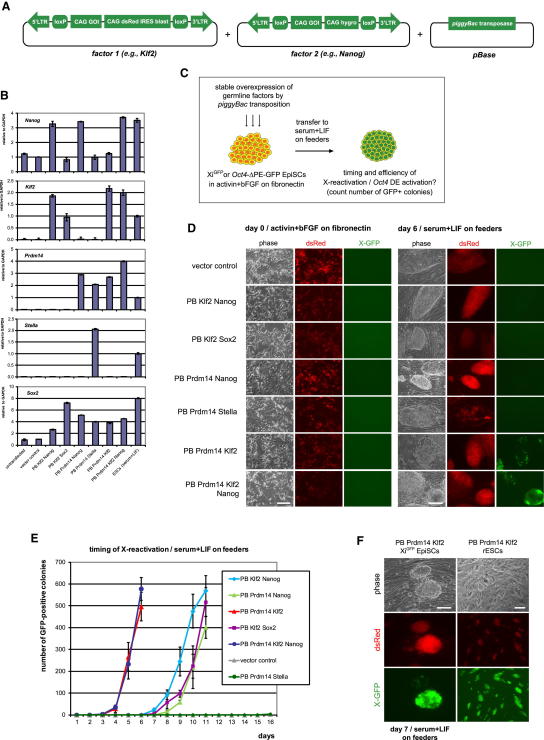
Germline Factors with Impact on Reprogramming and X Reactivation (A) *PiggyBac* (PB) constructs for the generation of epiSC lines with stable overexpression of germline genes. The PB vector with *loxP* sites, a dsRed reporter, and a linked antibiotic resistance gene allows for transgene excision by Cre expression. (B) Q-PCR analysis of transgene expression in Xi^GFP^ epiSCs with stable overexpression of germline genes in activin and bFGF relative to GAPDH. Error bars are mean ± SD (n = 2). (C) Experimental approach to identify germline factors affecting X reactivation. Stable PB epiSC lines with Xi^GFP^ or *Oct4*-ΔPE-GFP reporter are cultured in activin and bFGF and transferred to serum and LIF on feeder cells, and the number of GFP-positive colony patches is counted from 10,000/30,000/50,000 plated cells per 6-well every day. (D) Fluorescence images of PB Xi^GFP^ epiSCs overexpressing germline gene combinations in activin and bFGF (day 0) and after transfer to serum and LIF (day 6). Scale bars represent 100 μm. (E) Quantification of the timing of X reactivation after transfer of PB Xi^GFP^ epiSCs to serum and LIF. Data are shown as mean ± SD of three biological replicates from 50,000 plated cells/6-well. (F) Fluorescence images of Xi^GFP^ epiSCs overexpressing *Prdm14-Klf2* on day 7 in serum and LIF. The GFP-positive colony was picked and expanded as reverted ESCs (rESCs). Scale bars represent 100 μm. See also Figure S1.

Initially, we found that transient transfection of germline gene combinations did not induce Xi^GFP^ reporter expression in epiSCs (data not shown). We therefore generated epiSCs with stable expression of combinations of two or three candidate factors by *piggyBac* (PB) transposition (Figure 2A; Guo et al., 2009), starting with *Prdm14*, *Stella, Klf2, Nanog*, and *Sox2*. EpiSCs with stable expression of these factors at similar or higher levels than the endogenous transcripts in female X-GFP ESCs (Figure 2B) had increased Stella, Nanog, and Sox2 protein compared to vector control epiSCs (Figure S1B). The levels of *Prdm14* in manipulated epiSCs were higher than those in ESCs cultured in serum and LIF (Figure 2B), but similar to the levels in ESCs maintained in 2i (Ying et al., 2008) and LIF, with the latter having about four times higher levels of *Prdm14* transcript (Figure S1C). Transcript and protein levels of Sox2, a direct Prdm14 target (Ma et al., 2011), showed an increase in epiSCs with a gain of function for *Prdm14* (Figures 2B and S1E), but there was no change in *Nanog* (Figure 2B). Despite these changes, the X-GFP reporter remained repressed, indicating stability of the Xi (Figure 2D, left panel).

To exclude a possibility that X chromosome reactivation had been initiated but did not proceed to biallelic expression of X-linked genes, we analyzed the levels of *Xist* and *Tsix* in epiSCs sorted for SSEA1 to eliminate any differentiated cells (Hayashi and Surani, 2009b). Quantitative real-time PCR (Q-PCR) for *Xist* and *Tsix* showed that they remained highly expressed and fully repressed, respectively (Figure S1D). Moreover, the epiSCs continued to display nuclear H3K27me3 domains (Figure S1E), which further confirmed that the Xi was not reactivated.

Thus, our data show that X inactivation is remarkably stable in epiSCs expressing combinations of germline factors, which indicates that LIF-Stat3 signaling may be required to trigger reprogramming and X reactivation (Bao et al., 2009; Yang et al., 2010).

### Transfer to LIF-Stat3 Reveals Reprogramming Potential of Germline Factors

Next, we examined the impact of LIF and serum (henceforth called LIF-Stat3) on Xi^GFP^ expression during reversion of epiSCs to ESCs on feeder cells (Figure 2C; Bao et al., 2009). By counting the number of GFP-positive colony patches every day, we found that epiSCs with exogenous *Prdm14* and *Stella* or vector control did not show activation of the X-GFP reporter despite culture for 2 weeks in LIF-Stat3 (Figures 2D and 2E). In contrast, epiSCs expressing *Klf2* and *Nanog* or *Prdm14* and *Nanog* showed GFP expression after 7–8 days (Figure 2E). However, epiSCs expressing *Prdm14* and *Klf2* produced GFP-positive colonies after just 3–4 days, resulting in about 500 GFP-positive colonies from 50,000 plated cells on day 6 (Figures 2D and 2E). This effect is striking considering that X reactivation is a late event during reprogramming (Stadtfeld et al., 2008). The addition of *Nanog* had no additional impact (Figure 2E), although *Prdm14* and *Nanog* have been proposed to cooperate in ESCs (Ma et al., 2011).

Because *Prdm14* activates *Sox2* (Ma et al., 2011; Yamaji et al., 2008), we asked whether epiSCs with exogenous *Sox2* and *Klf2* together could also accelerate activation of the X-GFP reporter, but this combination resulted in a slower response compared with *Prdm14* and *Klf2* (Figure 2E). This suggests that other critical targets are activated or repressed by *Prdm14* and might have a role during reversion to ESCs.

Initially, epiSCs with *Prdm14* and *Klf2* transferred to LIF-Stat3 showed mosaic GFP expression (Figure S1F), but the GFP signal frequently spread out to the whole colony (Figure 2F). These GFP-positive colonies were picked and expanded, resulting in lines with homogeneous GFP expression (Figure 2F), which indicates a reactivated X chromosome.

Thus, our data show that *Prdm14* and *Klf2* trigger particularly rapid X reactivation in epiSCs upon transfer to LIF-Stat3, raising the possibility of cooperation between the two factors.

### *Prdm14* Is Not Sufficient but Acts Synergistically with *Klf2* to Accelerate X Reactivation

**Figure 3:**
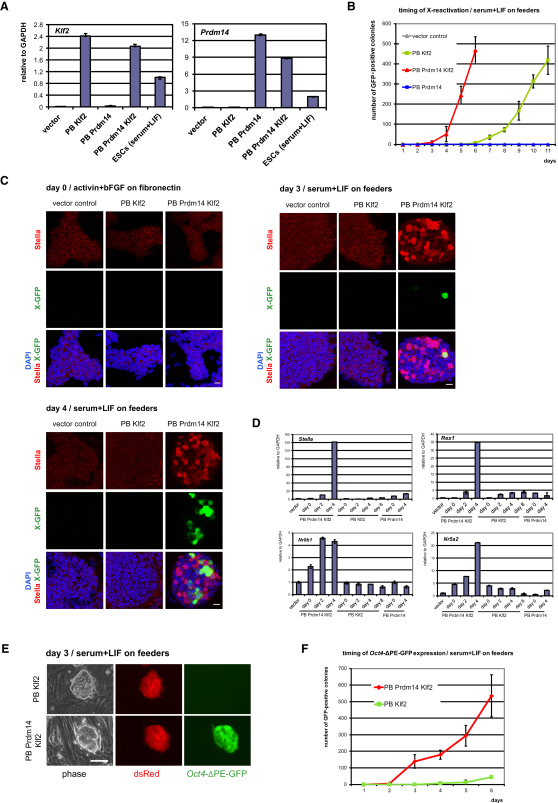
*Prdm14* Synergizes with *Klf2* to Accelerate Reprogramming (A) Q-PCR analysis of *Prdm14* and *Klf2* transgene expression in Xi^GFP^ epiSCs with stable overexpression of *Klf2*, *Prdm14*, *Prdm14-Klf2*, or vector control in activin and bFGF relative to GAPDH. Error bars are mean ± SD (n = 2). (B) Quantification of the timing of X reactivation after transfer of Xi^GFP^ epiSCs with overexpression of *Klf2*, *Prdm14*, *Prdm14-Klf2*, or vector control to serum and LIF. Data are shown as mean ± SD of three biological replicates from 50,000 plated cells/6-well. (C) Double immunostaining for Stella and X-GFP in Xi^GFP^ epiSCs overexpressing *Klf2*, *Prdm14-Klf2*, or vector control in activin and bFGF (day 0) and after transfer to serum and LIF (day 3, day 4). Nuclei were stained with DAPI. Scale bars represent 20 μm. (D) Q-PCR analysis for *Stella*, *Rex1*, *Nr0B1*, and *Nr5a2* expression in Xi^GFP^ epiSCs with *Klf2*, *Prdm14*, *Prdm14-Klf2*, or vector control in activin and bFGF (day 0) and after transfer to serum and LIF (day 2/4/8). Data are shown relative to GAPDH and error bars are mean ± SD (n = 2). (E) Fluorescence images of *Oct4*-ΔPE-GFP epiSCs with *Klf2 ± Prdm14* on day 3 after transfer to serum and LIF. Scale bar represents 100 μm. (F) Quantification of the timing and efficiency of *Oct4* distal enhancer activation after transfer of *Oct4*-ΔPE-GFP epiSCs with *Klf2 ± Prdm14* to serum and LIF. Data are shown as mean ± SD of three biological replicates from 10,000 plated cells/6-well. See also Figures S2 and S3.

To test whether *Prdm14* and *Klf2* cooperate to promote reprogramming, we examined Xi^GFP^ epiSCs expressing these genes individually or together (Figure 3A). Prior to transfer to LIF-Stat3, Xi^GFP^ epiSCs with different factor combinations retained a characteristic epiSC gene expression profile, with low expression of *Stella*, *Rex1*, *Nr0B1*, and *Tsix* and high expression of *Xist* and *Fgf5* (Figure S2A). Importantly, epiSCs with *Prdm14* alone did not show Xi^GFP^ reporter expression after 11 days in LIF-Stat3 (Figure 3B), and even after 21 days, only a slight effect was seen (Figure S2B). This indicates that *Prdm14* alone is not sufficient to induce a major effect on reprogramming and X reactivation. By contrast, epiSCs with *Klf2* alone produced GFP-positive cells but in a relatively protracted manner on day 6–7, and more robustly on day 9, compared to a highly accelerated rate of reprogramming by *Prdm14* and *Klf2* (Figure 3B). This demonstrates a combinatorial role for *Prdm14* and *Klf2* in X reactivation. The difference in the timing of X reactivation was not due to faster division of epiSCs carrying *Prdm14* and *Klf2* compared to cells with *Klf2* alone, as monitored by growth curves (Figure S2C), although any putative contribution of cell division to X reactivation will require tracking the number of cell divisions in real-time.

Therefore, although *Prdm14* does not promote reprogramming on its own, it synergizes with *Klf2* to trigger particularly rapid X reactivation.

### *Prdm14* and *Klf2*: A Potent Combination for Epigenetic Reprogramming

We noticed that Xi^GFP^ epiSCs that express *Prdm14* and *Klf2* formed compact, dome-shaped colonies faster than cells expressing *Klf2* alone when cultured in LIF-Stat3 (Figure S2D). Indeed, Stella, which is expressed in ESCs and PGCs (Payer et al., 2006), was induced on day 3 in LIF-Stat3 when both *Prdm14* and *Klf2* were present, but not in cells with *Klf2* alone or vector control (Figure 3C). In epiSCs with *Prdm14-Klf2*, X-GFP-positive cells appeared within Stella-expressing colonies (Figure 3C), suggesting that Stella expression may precede X reactivation, consistent with the temporal sequence observed in PGCs (Chuva de Sousa Lopes et al., 2008). After 4 days in LIF-Stat3, larger clusters of X-GFP-positive cells were located within Stella-positive colonies (Figure 3C). However, their expression did not always coincide (Figure 3C), which could be due to heterogeneity and dynamic changes in Stella expression in ESCs (Hayashi et al., 2008). Q-PCR analysis confirmed specific *Stella* induction in epiSCs with *Prdm14* and *Klf2* on day 4 in LIF-Stat3, in contrast to cells expressing *Klf2* alone, *Prdm14* alone, or vector control (Figure 3D). Bisulfite sequencing revealed partial DNA demethylation of the *Stella* locus in sorted GFP-positive cells from these cultures on day 4 in LIF-Stat3 (Figure S2E). Furthermore, *Rex1*, *Nr0B1*, and *Nr5a2* were induced earlier, when both *Prdm14* and *Klf2* were expressed in epiSCs (Figure 3D). These data suggest that *Prdm14* and *Klf2* together accelerate epigenetic reprogramming in epiSCs.

To gain further insight on the specificity of the response of epiSCs to *Prdm14* and *Klf2,* we examined the response to other Klf family members, namely *Klf4* and *Klf5,* that can promote reprogramming of fibroblasts to iPSCs (Nakagawa et al., 2008); both are expressed in ESCs, although only *Klf5* is detected in PGCs (Jiang et al., 2008; Kurimoto et al., 2008). We found that although *Klf2, Klf4*, or *Klf5* alone could induce reprogramming of epiSCs (Hall et al., 2009), the efficiency of the process was higher when *Prdm14* was also present (Figure S2F). However, *Prdm14* was most potent when combined with *Klf2*, compared to the response with either *Klf4* or *Klf5* (Figure S2F) or with other pluripotency factors such as *Nanog* (Figure S2G). The specific effect of *Prdm14* with *Klf2* is unexpected, because *Klf2, Klf4*, and *Klf5* have redundant functions in mouse ESCs (Jiang et al., 2008). This suggests that re-entry into naive pluripotency may require different factors or factor combinations than maintenance of a naive pluripotent state.

### Fast and Efficient *Oct4* Enhancer Switch by *Prdm14* and *Klf2*

To further determine the rate and efficiency of reprogramming of epiSCs by *Prdm14* and *Klf2*, we monitored it with the *Oct4-*ΔPE-GFP reporter, which contains *Oct4* DE only. This reporter is repressed in epiSCs, but it is activated in the course of reversion to ESCs and during PGC specification (Bao et al., 2009). We introduced *Prdm14* and *Klf2* in *Oct4-*ΔPE-GFP epiSCs and found that this reporter was also repressed in cells cultured in activin and bFGF (Figures S3A and S3B). However, upon transfer to LIF-Stat3, we detected an extremely rapid and efficient activation of the reporter, with GFP-positive cells appearing as early as day 2 and with an efficiency of reporter activation of 5% after 6 days in LIF-Stat3 (Figures 3F and S3C). The speed of the response in the presence of *Klf2* alone did not match with that observed with *Prdm14-Klf2* (Figures 3E and 3F). These GFP-positive cells, called reverted ESCs (rESCs), could be maintained thereafter and exhibit homogeneous *Oct4*-ΔPE-GFP expression (Figure S3D). Thus, these data further suggest that *Prdm14-Klf2* trigger particularly fast and efficient epigenetic reprogramming. Interestingly, *Klf2* expression follows *Prdm14* induction in early germ cells (Figure S3E; Kurimoto et al., 2008), suggesting that the two factors may also act similarly to promote reprogramming of PGCs. Indeed, the combination of *Prdm14-Klf2* was particularly potent for induction of PGC-like cells, which is not seen with wild-type epiSCs (Figures S3F and S3G; Hayashi et al., 2011; Hayashi and Surani, 2009b) or when the two factors are tested individually. Additional factors or factor combinations may be identified in the future that may further enhance PGC specification.

To exclude the possibility that the effect of *Prdm14-Klf2* is mediated through factors present in serum or secreted by feeder cells, we examined reprogramming in serum- and feeder-free 2i/LIF conditions (Ying et al., 2008). Reprogramming by *Prdm14* and *Klf2* was again more efficient compared with *Klf2* alone, resulting in more compact GFP-positive colonies on day 6–9 (Figures S3H–S3J). There was also specific induction of *Stella*, *Rex1*, and *Nr5a*2 as early as day 2 (Figure S3K). These observations suggest that *Prdm14* and *Klf2* enhance epigenetic reprogramming also upon Erk inhibition in serum- and feeder-free 2i/LIF conditions.

### EpiSCs Reprogrammed by *Prdm14* and *Klf2* Progress to Naive Pluripotency

**Figure 4:**
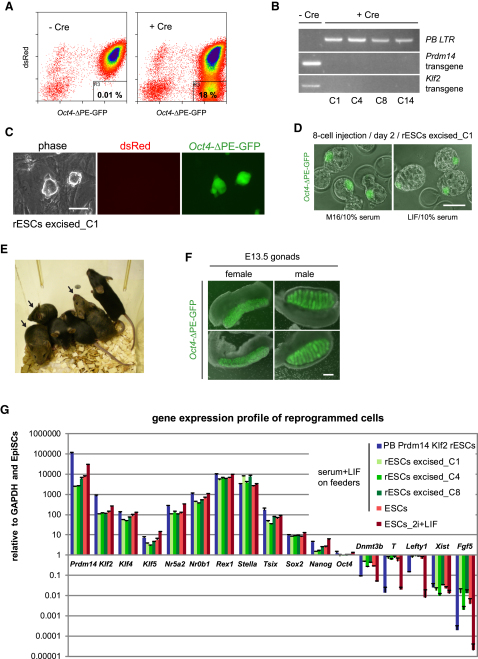
*Prdm14* and *Klf2* Reprogram EpiSCs to Naive Pluripotency (A) Flow cytometry analysis of dsRed expression in *Oct4*-ΔPE-GFP rESCs generated from *Prdm14-Klf2*-overexpressing epiSCs 3 days after transient Cre transfection. (B) Genomic PCR showing loss of *Prdm14* and *Klf2* transgenes and gain of recombined *PB LTR* fragments in four *Oct4*-ΔPE-GFP rESC clones. (C) Phase contrast and fluorescence images of *Oct4*-ΔPE-GFP rESCs cultured in serum and LIF. Scale bar represents 100 μm. (D) Inner cell mass contribution of *Oct4*-ΔPE-GFP rESCs 2 days after injection into E2.5 C57BL/6 morulae. Scale bar represents 100 μm. (E) Coat color chimera generated by injection of *Oct4*-ΔPE-GFP rESCs into C57BL/6 blastocysts. (F) Contribution of *Oct4*-ΔPE-GFP rESCs to E13.5 C57BL/6 male and female genital ridges. Scale bar represents 100 μm. (G) Q-PCR analysis for selected ESC and epiSC markers in *Oct4*-ΔPE-GFP rESCs before and after transgene excision and in *Oct4*-ΔPE-GFP ESCs cultured in LIF and serum or 2i. Data are shown relative to GAPDH and *Oct4*-ΔPE-GFP epiSCs. Error bars are mean ± SD (n = 2). See also Figure S4.

To confirm complete reprogramming of rESCs, we tested their potential to contribute to somatic lineages. The cells contributed to chimeras after transgene excision (Figures 4A and 4B) and not before, presumably because the PB transgenes were not silenced (Guo et al., 2009). These *Oct4*-ΔPE-GFP-positive rESCs (Figure 4C) had a similar gene expression profile to ESCs (Figure 4G), integrated into the inner cell mass upon 8-cell injection (Figure 4D), and contributed to coat-color chimeras (Figure 4E) and to E13.5 genital ridges (Figure 4F). Interestingly, rESCs with *Prdm14* and *Klf2* transgenes had lower transcript levels of *Dnmt3b*, *T*, *Lefty1*, and *Fgf5* than ESCs cultured in serum and LIF but similar levels as ESCs maintained in 2i and LIF (Figure 4G). Therefore, *Prdm14* and *Klf2* seem to generate rESCs that acquire a naive pluripotent state with lower levels of differentiation genes, which may have similarities with ESCs cultured in 2i. We confirmed the loss of nuclear foci of *Xist*, Ezh2, and H3K27me3 in rESCs (Figures S4A–S4C), indicating a fully reactivated X chromosome.

Hence, our data suggest that these rESCs have acquired a naive pluripotent state that remains stable in the absence of ectopic transgene expression.

### *Prdm14* Enhances Competence for Reprogramming

**Figure 5:**
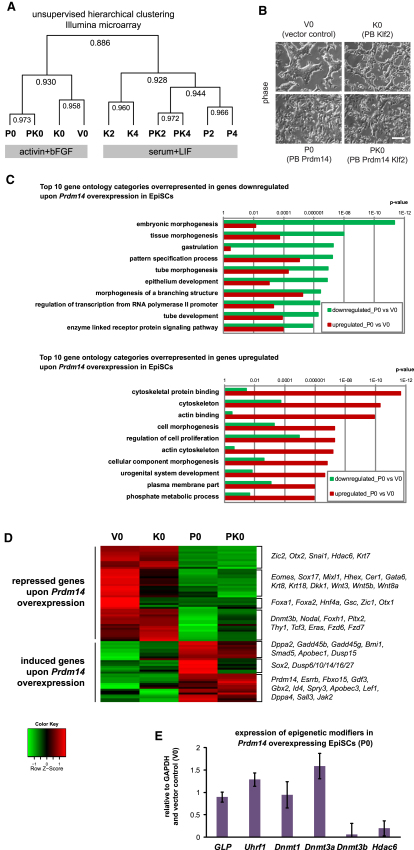
Gene Expression Changes upon *Prdm14* Overexpression in EpiSCs (A) Dendrogram derived from unsupervised hierarchical clustering of Illumina microarray samples with Pearson correlation coefficients: Xi^GFP^ epiSCs overexpressing *Klf2* (K0), *Prdm14* (P0), *Prdm14* and *Klf2* (PK0), or vector control (V0) in activin and bFGF; Xi^GFP^ epiSCs overexpressing *Klf2* (K2, K4), *Prdm14* (P2, P4), or *Prdm14* and *Klf2* (PK2, PK4) on day 2 and day 4 after transfer to serum and LIF. (B) Phase contrast images of Xi^GFP^ epiSCs overexpressing *Klf2, Prdm14*, *Prdm14* and *Klf2,* or vector control in activin and bFGF. Scale bar represents 100 μm. (C) Top 10 DAVID gene ontology categories overrepresented in down- or upregulated genes upon *Prdm14* overexpression in epiSCs compared to vector control in activin and bFGF (FDR < 0.005). (D) Heatmap showing selected down- or upregulated genes upon *Prdm14* overexpression in epiSCs compared to vector control in activin and bFGF (FDR < 0.005). (E) Q-PCR analysis for expression of epigenetic modifiers in epiSCs overexpressing *Prdm14* in activin and bFGF relative to GAPDH and vector control. Error bars are mean ± SD (n = 2). See also Figures S5 and S6 and Tables S1 and S2.

To gain insight into the mechanism of acceleration of reprogramming by *Prdm14* and *Klf2,* we analyzed the global gene expression changes, starting with epiSCs cultured in activin and bFGF, and after 2–4 days in LIF-Stat3 culture (Figures 5A and S5A). Unsupervised hierarchical clustering revealed two main clusters, corresponding to epiSCs in activin and bFGF, and after culture in LIF-Stat3, respectively (Figure 5A). Strikingly, epiSCs maintained in activin and bFGF with *Prdm14* alone were similar to cells with both *Prdm14* and *Klf2*, whereas epiSCs with *Klf2* alone clustered together with vector control. The effect of *Prdm14* is also reflected in the changes in epiSC morphology (Figure 5B).

We analyzed the transcriptome of epiSCs for additional changes induced by *Prdm14* and found that 1,433 genes were induced and 1,310 genes were repressed in epiSCs with *Prdm14* compared to vector control (FDR < 0.005; Table S1). Reanalysis of Prdm14 ChIP-Seq data for ESCs (Ma et al., 2011) revealed that 1,135/1,433 upregulated genes (p value 1.38 × 10^−26^) and 1,088/1,310 downregulated genes (p value 6.16 × 10^−42^) are targets of Prdm14 (Table S1), indicating a direct effect on the transcriptome and suggesting that Prdm14 can act both as an activator and a repressor. Gene ontology analysis showed that gastrulation, embryonic morphogenesis, and tissue morphogenesis genes were predominantly repressed, whereas cytoskeletal genes were induced (Figure 5C). We confirmed that genes associated with early lineage specification were repressed (Figure 5D) and many of them are Prdm14 targets, such as *Nodal, Foxa2, Gata6, Hhex, Eomes, Foxh1*, and *Otx2* (Ma et al., 2011). Whereas some genes were repressed in epiSCs with *Klf2*, most of them were repressed only when *Prdm14* was present (Figure 5D). Thus, whereas Prdm14 represses extraembryonic endoderm differentiation in ESCs (Ma et al., 2011), it may also reduce differentiation toward early somatic lineages in epiSCs. Indeed, we found that *Prdm14* overexpression inhibited differentiation of epiSCs into endoderm but not into neuroectoderm (Figure S6), which is consistent with the predominant repression of mesendodermal genes in activin and bFGF (Figure 5D).

Among the repressed genes in epiSCs by *Prdm14*, we also found *Thy1*, which is repressed during the early phase of somatic cell reprogramming (Stadtfeld et al., 2008); *Snai1*, a Prdm14 target and an effector of epithelial-to-mesenchymal transition (Samavarchi-Tehrani et al., 2010); *Tcf3*, a Prdm14 target and a negative regulator of pluripotency (Guo et al., 2011); and components of the Wnt pathway (Figure 5D).

Notably, several early epiblast or ESC-associated genes, such as *Sox2, Gbx2, Esrrb, Fbxo15, Gdf3, Dppa2*, and *Dppa4* as well as the FGF inhibitor *Spry3* and several members of the dual specificity phosphatase (*Dusp*) family, were induced (Figure 5D); *Dppa4* is particularly interesting, because its levels correlate with the efficiency of epiSC reversion (Han et al., 2010). Altogether, these data suggest that Prdm14 enables repression of early germ layer-associated genes as well as induction of early epiblast markers in epiSCs.

Next, we compared the reciprocal effects of gain of function of *Prdm14* in epiSCs with *Prdm14* knockdown in ESCs (Ma et al., 2011). We found only 37/1,443 induced genes, such as *Sox2*, repressed upon *Prdm14* knockdown, and 94/1,310 repressed genes, such as *Dnmt3b* and *Snai1*, induced (Table S2). The limited overlap in gene expression changes may suggest cell context-dependent effects of Prdm14.

Among the epigenetic modifier targets, we found that Prdm14 repressed *Dnmt3b* and *Hdac6* in epiSCs (Figures 5D and 5E). *Dnmt3b,* which is predominantly responsible for DNA methylation in postimplantation epiblast cells (Borgel et al., 2010), is also repressed in PGCs (Yabuta et al., 2006). However, we did not detect changes in *GLP* (*Ehmt1*) and *Uhrf1* (Figure 5E) that are efficiently repressed in early PGCs (Kurimoto et al., 2008); *GLP* was suggested to be responsible for the genome-wide decrease in H3K9me2 (Hajkova et al., 2008). Consistently, there was no decrease in H3K9me2 levels in epiSCs with gain of *Prdm14* (Figure S5B). Nonetheless, there was rapid DNA demethylation in epiSCs during reprogramming as judged by re-expression of *Stella* and *Rex1* (Figures 3C and 3D), which are methylated in epiSCs (Bao et al., 2009).

A particularly interesting observation was that many differentiation-associated genes, which were repressed in epiSCs with *Prdm14*, remained highly expressed or were even induced in epiSCs with *Klf2* when cultured in LIF-Stat3 (Figure S5C). Thus, although *Klf2* in epiSCs may induce differentiation upon transfer to LIF-Stat3, such tendency is blocked efficiently by *Prdm14* and may contribute to fast and efficient reprogramming.

Therefore, Prdm14 may enhance the competence for reprogramming by repression of differentiation and induction of early epiblast markers.

### Prdm14 Enhances Klf2 Recruitment to Target Loci in LIF-Stat3

**Figure 6:**
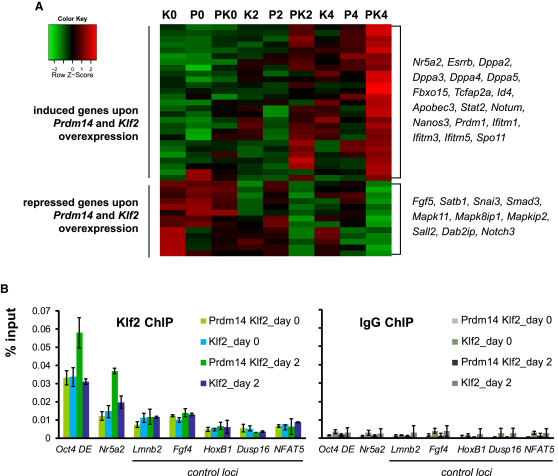
Early Gene Expression Dynamics during EpiSC Reprogramming by *Prdm14* and *Klf2* (A) Heatmap of selected genes that are up- or downregulated specifically upon overexpression of *Prdm14* and *Klf2* compared to the factors overexpressed individually upon transfer to serum and LIF (FDR < 0.005). (B) ChIP analysis for Klf2 on Klf2+Prdm14-bound targets *Oct4* DE and *Nr5a2* in epiSCs overexpressing *Prdm14-Klf2* or *Klf2* alone in activin and bFGF (day 0) and 2 days after transfer to serum and LIF (day 2). Data were normalized to input and are shown as mean ± SD of three biological replicates. See also Figure S7 and Tables S3 and S4.

The extensive gene expression changes induced by *Prdm14* alone in epiSCs, although surprising, were not sufficient for successful reprogramming in LIF-Stat3, suggesting that a combinatorial role of *Prdm14-Klf2* is critical, which is reflected in the gene expression changes (Figure 6A; Table S3). Gene ontology analysis revealed that among genes specifically induced with *Prdm14-Klf2* were transcription factors, DNA binding, and negative regulation of differentiation genes (Figure S7A). Among the upregulated genes were reprogramming and ESC-associated genes, such as *Nr5a2, Esrrb, Dppa2/3/4/5, Fbxo15*, and *Tcfap2a* (Figure 6A). The downregulated genes included those involved in gastrulation, primary germ layer formation, and tissue morphogenesis (Figure S7A), such as the epiblast marker *Fgf5* and the X-inactivation regulator *Satb1* (Figure 6A; Agrelo et al., 2009).

Reanalysis of ChIP-Seq data for Prdm14 (Ma et al., 2011) and Stat3 (Chen et al., 2008) and ChIP-on-chip for Klf2 (Jiang et al., 2008) showed that 578/611 Klf2-bound genes (95%) were also bound by Prdm14, whereas 238 of them (39%) are bound by both Prdm14 and Stat3 (Figure S7B and Table S4). Among Prdm14-Klf2-bound genes were the reprogramming factor *Nr5a2* (Guo and Smith, 2010) and *Oct4* DE (Figure S7C). To test whether Prdm14 might help to recruit Klf2 to these targets, we performed ChIP for Klf2 on the *Nr5a2* promoter and *Oct4* DE. This revealed increased Klf2 recruitment in epiSCs in the presence of *Prdm14* and *Klf2,* compared to cells with *Klf2* alone on day 2 of culture in LIF-Stat3 (Figure 6B), indicating that Prdm14 may promote the binding of Klf2 to enhance reprogramming.

### Does Reprogramming of EpiSCs to rESCs by *Prdm14-Klf2* Require a Transition through a Blimp1-Positive Germ Cell-like State?

**Figure 7:**
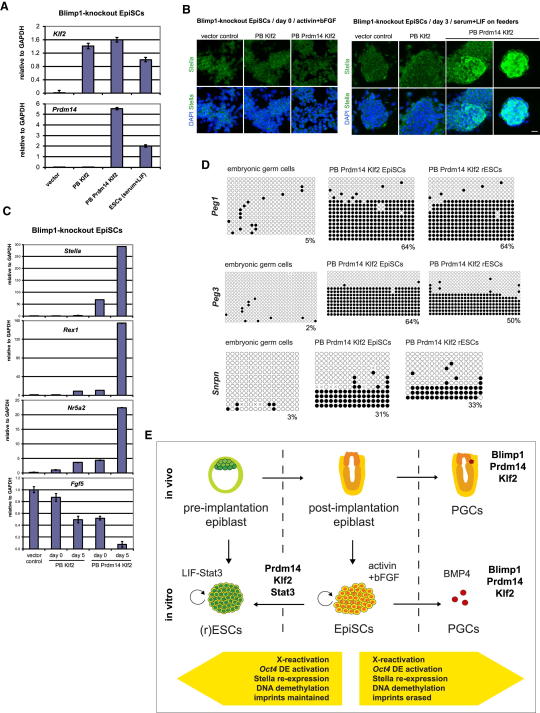
Reprogramming of Blimp1-Knockout EpiSCs by *Prdm14* and *Klf2* (A) Q-PCR analysis of *Prdm14* and *Klf2* transgene expression in Blimp1-knockout epiSCs overexpressing *Prdm14-Klf2, Klf2* alone, or vector control in activin and bFGF relative to GAPDH. Error bars are mean ± SD (n = 2). (B) Immunostaining for Stella in Blimp1-knockout epiSCs overexpressing *Prdm14-Klf2, Klf2* alone, or vector control in activin and bFGF (day 0) and after transfer to serum and LIF (day 3). Nuclei were stained with DAPI. Scale bar represents 20 μm. (C) Q-PCR analysis for *Stella*, *Rex1*, *Nr5a2*, and *Fgf5* expression in Blimp1-knockout epiSCs overexpressing *Prdm14-Klf2, Klf2* alone, or vector control in activin and bFGF (day 0) and after transfer to serum and LIF (day 5). Data are shown relative to GAPDH and error bars are mean ± SD (n = 2). (D) Bisulfite sequencing of *Peg1-*, *Peg3-*, and *Snrpn*-imprinted loci in embryonic germ cells derived from E11.5 PGCs as well as *Oct4*-ΔPE-GFP epiSCs and rESCs with *Prdm14-Klf2* overexpressed. CpG dinucleotides are shown as open (unmethylated) or filled (methylated) circles. (E) Schematic of epiSC and PGC reprogramming by *Prdm14-Klf2*.

We found that *Prdm14* and *Klf2* induced several germline-associated genes in epiSCs after transfer to LIF-Stat3, most notably *Blimp1* (*Prdm1*), *Stella* (*Dppa3*), *Fragilis* (*Ifitm1/3/5*), and *Nanos3* (Figure 6A), indicating that epiSC reprogramming by *Prdm14-Klf2* may require progression through a germ cell intermediate. If so, this process might be restricted in the absence of Blimp1, a crucial determinant of PGC specification (Ohinata et al., 2005; Vincent et al., 2005). We tested this hypothesis by using Blimp1-knockout epiSCs in our assay, but found that reprogramming was not affected as shown by the earlier appearance of Stella-expressing colonies, as well as specific induction of *Rex1* and *Nr5a2* and repression of *Fgf5* in cells with *Prdm14-Klf2* compared to those with *Klf2* alone (Figures 7A–7C). In addition, rESCs reprogrammed by *Prdm14* and *Klf2* had retained DNA methylation in the imprinted loci *Peg1*, *Peg3*, and *Snrpn* (Figure 7D).

Therefore, *Prdm14* and *Klf2* promote X reactivation and DNA demethylation (excepting loss of imprints), which also occurs in PGCs. However, *Prdm14-Klf2* may act to induce characteristics of naive pluripotency without necessarily passing through a Blimp1-positive germ cell intermediate. This function may be conserved not only in the reversion of epiSCs to rESCs but also in re-establishing a primary background of pluripotency in PGCs, which is vital to this lineage (Figure 7E; Yamaji et al., 2008).

## Discussion

Blimp1, Prdm14, and their targets drive PGC specification and reprogramming, which culminates in an epigenetic ground state (Surani and Hajkova, 2010). With an epiSC-based system to explore the role of germline factors in epigenetic reprogramming, we discovered a powerful synergistic effect of *Prdm14-Klf2* that accelerates and enhances reprogramming of epiSCs to rESCs. Notably, *Prdm14* alone has little effect on epiSC reprogramming, but it potentiates the action of *Klf2*; neither *Klf4* nor *Klf5* have an equivalent effect when combined with *Prdm14*.

### *Prdm14* Primes EpiSCs for Reprogramming

Whereas *Prdm14* accelerates reprogramming of epiSCs by *Klf2*, it has surprisingly little effect by itself, which suggests that *Prdm14* might prime epiSCs for reprogramming. Indeed, we show global changes in the transcriptome of epiSCs in response to *Prdm14* alone, which include induction of *Esrrb* and repression of *Tcf3* and *Nodal*. *Prdm14* apparently induces changes in epiSCs toward an earlier postimplantation epiblast-like state, with the induction of *Dppa4* (Han et al., 2010), which might contribute to the effectiveness of *Klf2* in the process. In addition, by repressing early lineage-specification genes, *Prdm14* may decrease the propensity of epiSCs toward heterogeneity (Han et al., 2010) and increase their responsiveness to LIF-Stat3 signaling and reprogramming factors. Prdm14, which binds predominantly to distal regulatory elements (Ma et al., 2011), might also be important for priming by inducing a permissive state in enhancers in preparation for interaction with the cognate promoters.

### *Prdm14* Promotes Klf2 Recruitment to Specific Targets

*Prdm14* enhances recruitment of Klf2 to key loci, such as *Nr5a2* and the distal *Oct4* enhancer, suggesting cooperation between the two factors. The binding sites of Prdm14 and Klf2 are in close proximity on these targets, suggesting that Klf2 could be recruited directly by Prdm14. However, because Prdm14 predominantly binds far away from the transcription start site (Ma et al., 2011), it might bring distal regulatory elements in close proximity to promoters; the resulting changes in the chromatin topography and potential chromatin-modifying activities of Prdm14, potentially through its PR/SET domain, may promote its combinatorial role with Klf2. Because the current analysis of Klf2 binding sites was performed with a tiling array interrogating only about 400 genes (Jiang et al., 2008), the full extent of the overlap of binding between Prdm14 and Klf2 remains unknown and the combinatorial effect could involve cobinding on genes outside the scope of that assay. Therefore, genome-wide binding profiles of Klf2 in the absence and presence of Prdm14 during epiSC reversion are needed to identify Klf2 targets that are Prdm14 dependent and independent. Notably, reprogramming occurs only after transfer of epiSCs to LIF-Stat3, indicating a functional interaction of Stat3 with Prdm14 and/or Klf2.

### The Impact of *Prdm14-Klf2* on X Reactivation and DNA Demethylation

The epiSC-based assay we describe exhibits a rapid effect on X reactivation as seen in early germ cells (Chuva de Sousa Lopes et al., 2008). *Prdm14-Klf2* can potentially act to regulate the expression of *Xist* and *Tsix*; Prdm14 itself binds directly to *Xist* intron 1 and to *Rnf12*, an X-linked *Xist* activator (Jonkers et al., 2009; Ma et al., 2011), whereas Klf2 might promote *Tsix* expression resulting in repression of *Xist* (Navarro et al., 2010). Although Oct4, Sox2, and Nanog are not enriched on intron 1 of *Xist* in epiSCs, Prdm14 could promote recruitment of the pluripotency factors during reversion of epiSCs to rESCs, e.g., by altering the local status of the chromatin.

The derivation of epiSCs from epiblast is coupled with acquisition of DNA methylation on loci such as *Stella* and *Rex1* (Bao et al., 2009), which could contribute to the refractory nature of epiSC reprogramming. DNA demethylation by *Prdm14-Klf2* occurs rapidly after epiSCs are transferred to LIF-Stat3 as judged by the activation of *Stella* and *Rex1*. The rapid loss of DNA methylation cannot be easily accounted for by a passive mechanism, because expression of the maintenance methylase *Dnmt1* or its vital cofactor *Uhrf1* were unaffected; *Uhrf1* is repressed in PGCs (Kurimoto et al., 2008). Thus, rapid DNA demethylation might occur through alternative mechanisms, potentially involving hydroxylation of 5-methylcytosine by enzymes, such as Tet1 and Tet2, which are detected at the time of reprogramming in PGCs, and in ESCs (Ficz et al., 2011; Hajkova et al., 2010; Ito et al., 2011). A recent study indicates that *Stella* and *Rex1* are targets of Tet1/Tet2 (Ficz et al., 2011). Further studies are needed to determine the precise mechanism of DNA demethylation.

### Diverse Roles of Prdm14 in Mouse and Human Pluripotency

Besides the role of Prdm14 in PGCs (Yamaji et al., 2008), PRDM14 is obligatory for the maintenance of pluripotency in human ESCs, whereas Prdm14 is not expressed in epiSCs (Chia et al., 2010). Knockdown of PRDM14 induces human ESC differentiation (Chia et al., 2010), as also reported for mouse ESCs (Ma et al., 2011). Furthermore, PRDM14 binds to the proximal *Oct4* enhancer in human ESCs, whereas it is the distal enhancer that is bound by Prdm14 and gets activated during the reversion of epiSCs to rESCs (Chia et al., 2010; Ma et al., 2011). Notably, naive human ESCs, with two active X chromosomes, have recently been generated by introduction of KLF2 and KLF4 in 2i/LIF conditions (Hanna et al., 2010), and functional cooperation between PRDM14 and KLF2 should be investigated in this context.

### Reprogramming toward ESCs versus PGCs

*Prdm14*-*Klf2*-induced epigenetic changes in epiSCs during reversion to rESCs show some features in common with germ cell reprogramming, including X reactivation and DNA demethylation. The different cellular context, together with Blimp1 in PGCs, may be crucial for germ cell-specific features of reprogramming, especially the erasure of imprints. Indeed, we show that *Prdm14-Klf2*-induced reversion occurs efficiently in Blimp1-knockout epiSCs. In addition, Blimp1 is required neither for derivation and maintenance of ESCs nor for reversion of epiSCs to rESCs, but it is required for PGC specification (Ohinata et al., 2005; S.B., H.G. Leitch, M.A.S., et al., unpublished).

EpiSCs show a significant loss of competence for PGC specification compared with postimplantation epiblast or ESCs (Hayashi et al., 2011) and a poor ability to undergo reversion to rESCs (Bao et al., 2009; Guo et al., 2009). Studies similar to the effects of *Prdm14-Klf2* that promote both induction of PGC-like cells from epiSCs and reversion to rESCs could also advance our knowledge of the molecular basis of competence.

Cell culture-based systems that recapitulate events in early germ cells might be mutually informative for epigenetic reprogramming in general, while increasing our knowledge of the complex events in early germ cells that are vital for totipotency and a prerequisite for pluripotency in vivo.

## Experimental Procedures

Animal studies were authorized by a UK Home Office Project License and carried out in a Home Office-designated facility.

### EpiSC Reprogramming Assays in Serum and LIF

Pooled stable transfectants were plated at a density of 10,000/30,000/50,000 cells per well of a 6-well tissue culture plate in standard ESC medium supplemented with LIF (1000 U/ml; ESGRO; Chemicon) and 20% fetal bovine serum (GIBCO) on mitomycin C-treated MEFs. The medium was first replaced after 48 hr and subsequently every 24 hr. Unless otherwise indicated, the number of GFP-positive colony patches per well was counted every day with an Olympus IX71 inverted microscope and results are shown as mean ± standard deviation (SD) of three independent experiments.

## Accession Numbers

Microarray data are available from the ArrayExpress repository (http://www.ebi.ac.uk/arrayexpress) with the accession number E-MTAB-782.

## Supplemental Information

Document S1. Supplemental Experimental Procedures, Figures S1–S7, and Table S5

Table S1. List of Up- and Downregulated Genes upon *Prdm14* Overexpression in EpiSCs Compared to Vector Control

Table S2. Comparison of Gene Expression Changes upon *Prdm14* Overexpression in EpiSCs with *Prdm14* Knockdown in ESCs

Table S3. List of Up- and Downregulated Genes upon *Prdm14* and *Klf2* Overexpression in EpiSCs Compared to the Factors Overexpressed Individually upon Transfer to Serum and LIF on Feeder Cells

Table S4. Overlap of Published Klf2 ChIP-on-Chip Targets with Prdm14 and Stat3 ChIP-Seq Targets
