# Supplementary material for: Epiblast Stem Cell-Based System Reveals Reprogramming Synergy of Germline Factors
Source: Cell Stem Cell. 2012 Apr 6;10(4):425–39. doi: 10.1016/j.stem.2012.01.020 (PMC3343665; doi:10.1016/j.stem.2012.01.020)
Supplement: Document S1. Supplemental Experimental Procedures, Figures S1–S7, and Table S5 [file mmc1.pdf]

Immunity, Volume *36*

Supplemental Information

Epiblast Stem Cell-Based System Reveals Reprogramming Synergy of Germline Factors

Astrid Gillich, Siqin Bao, Nils Grabole, Katsuhiko Hayashi, Matthew W.B. Trotter, Vincent Pasque, Erna Magnúsdóttir, and M. Azim Surani

Supplemental Inventory:

SUPPLEMENTAL DATA

Figure S1 / related to Figures 1 and 2

Figure S2 / related to Figure 3

Figure S3 / related to Figure 3

Figure S4 / related to Figure 4

Figure S5 / related to Figure 5

Figure S6 / related to Figure 5

Figure S7 / related to Figure 6

Table S1 / related to Figure 5

Table S2 / related to Figure 5

Table S3 / related to Figure 6

Table S4 / related to Figure 6

Table S5 / Sequences of primers used in this study

(Tables S1-S4 are provided separately as Excel files)

SUPPLEMENTAL EXPERIMENTAL PROCEDURES

SUPPLEMENTAL REFERENCES

Figure S1

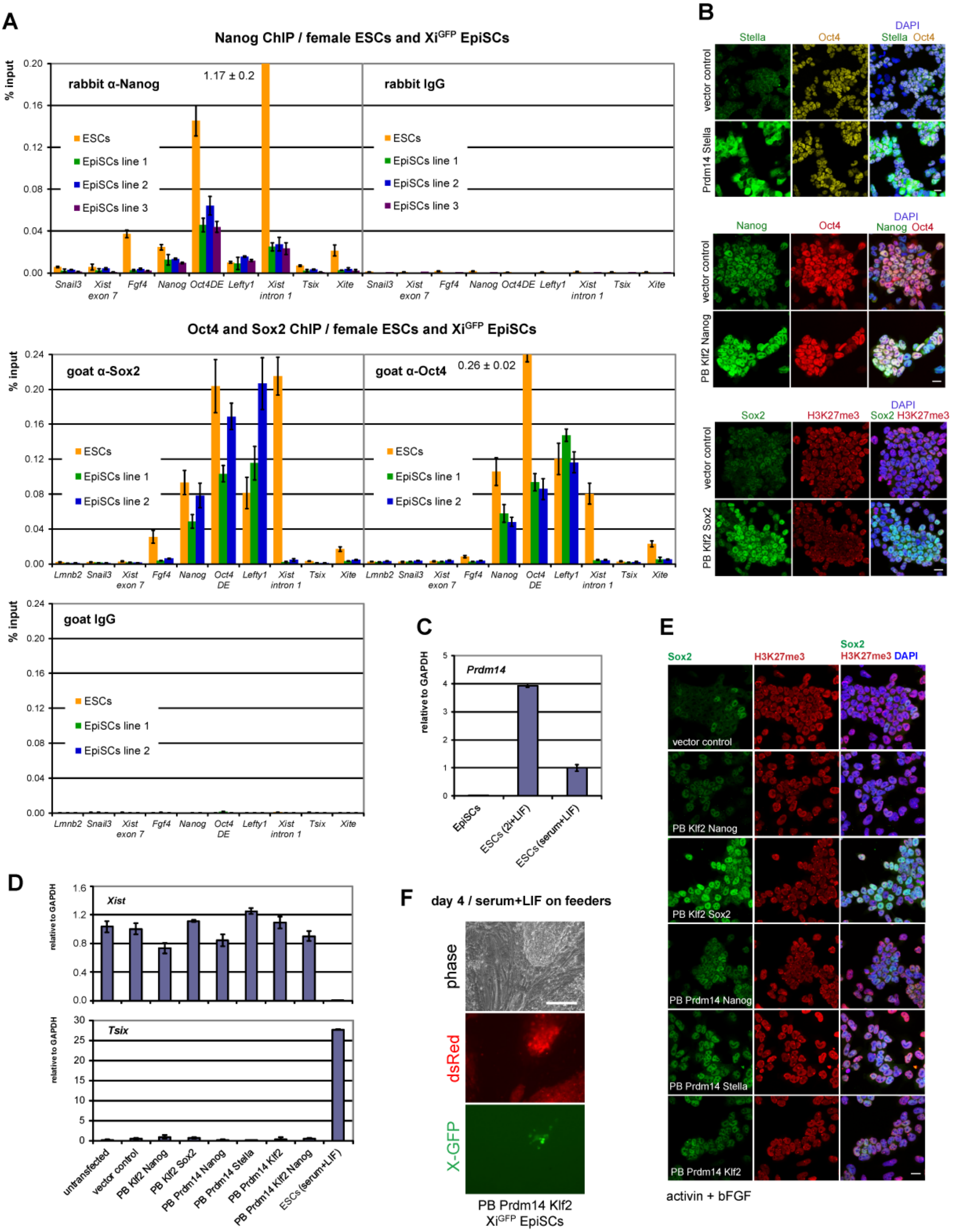

A
Nanog ChIP / female ESCs and Xi^GFP EpiSCs
% input
rabbit α-Nanog
1.17 ± 0.2
rabbit IgG
ESCs
EpiSCs line 1
EpiSCs line 2
EpiSCs line 3
0.20
0.16
0.12
0.08
0.04
0.00
Snail3
Xist exon 7
Fgf4
Nanog
Oct4DE
Lefty1
Xist intron 1
Tsix
Xite
Oct4 and Sox2 ChIP / female ESCs and Xi^GFP EpiSCs
goat α-Sox2
goat α-Oct4
0.26 ± 0.02
0.24
Lmnb2
Oct4 DE
goat IgG
B
Stella
Oct4
DAPI Stella Oct4
vector control
Prdm14 Stella
Nanog
DAPI Nanog Oct4
PB Klf2 Nanog
Sox2
H3K27me3
DAPI Sox2 H3K27me3
PB Klf2 Sox2
C
relative to GAPDH
Prdm14
EpiSCs
ESCs (2i+LIF)
ESCs (serum+LIF)
D
Xist
Tsix
untransfected
vector control
PB Klf2 Nanog
PB Klf2 Sox2
PB Prdm14 Nanog
PB Prdm14 Stella
PB Prdm14 Klf2
PB Prdm14 Klf2 Nanog
ESCs (serum+LIF)
E
Sox2
H3K27me3
Sox2 H3K27me3 DAPI
vector control
PB Klf2 Nanog
PB Klf2 Sox2
PB Prdm14 Nanog
PB Prdm14 Stella
PB Prdm14 Klf2
activin + bFGF
F
day 4 / serum+LIF on feeders
phase
dsRed
X-GFP
PB Prdm14 Klf2 Xi^GFP EpiSCs

Figure S1. Characterisation of EpiSCs with Stable Overexpression of Germ Line Factor Combinations, Related to Figures 1 and 2

(A) ChIP analysis for Oct4, Sox2 and Nanog on *Xist* intron 1 in Xi^{GFP} EpiSCs and female X-GFP ESCs. Genomic DNA was immunoprecipitated using anti-Oct4, anti-Sox2, anti-Nanog and rabbit/goat IgG antibodies and subjected to Q-PCR (see Table S5 for primer sequences). Data were normalised to input and are shown as mean±s.d. of 3 biological replicates.
(B) Immunostaining for Stella, Nanog, Oct4, Sox2 and H3K27me3 in PB EpiSCs with stable overexpression of combinations of germ line genes (commercially available Klf2 and Prdm14 antibodies did not work for immunostaining). Nuclei were stained with DAPI. Scale bars, 10 µm.
(C) Q-PCR analysis of *Prdm14* expression in Xi^{GFP} EpiSCs compared to X-GFP ESCs cultured in serum and LIF and in 2i and LIF relative to GAPDH. Error bars are mean±s.d. (n=2).
(D) Q-PCR analysis of *Xist* and *Tsix* expression in PB EpiSCs overexpressing candidate factor combinations relative to GAPDH. Cells were sorted for SSEA1 expression to eliminate any differentiated cells. Error bars are mean±s.d. (n=2).
(E) Double immunostaining for H3K27me3 and Sox2 in PB EpiSCs overexpressing candidate gene combinations. Nuclei were counterstained with DAPI. Scale bar, 10 µm.
(F) Phase contrast and fluorescence images of Xi^{GFP} EpiSCs overexpressing *Prdm14* and *Klf2* on day 4 after transfer to serum and LIF on feeder cells, showing the appearance of X-GFP-positive cells in a mosaic pattern. Scale bar, 100 µm.

Figure S2

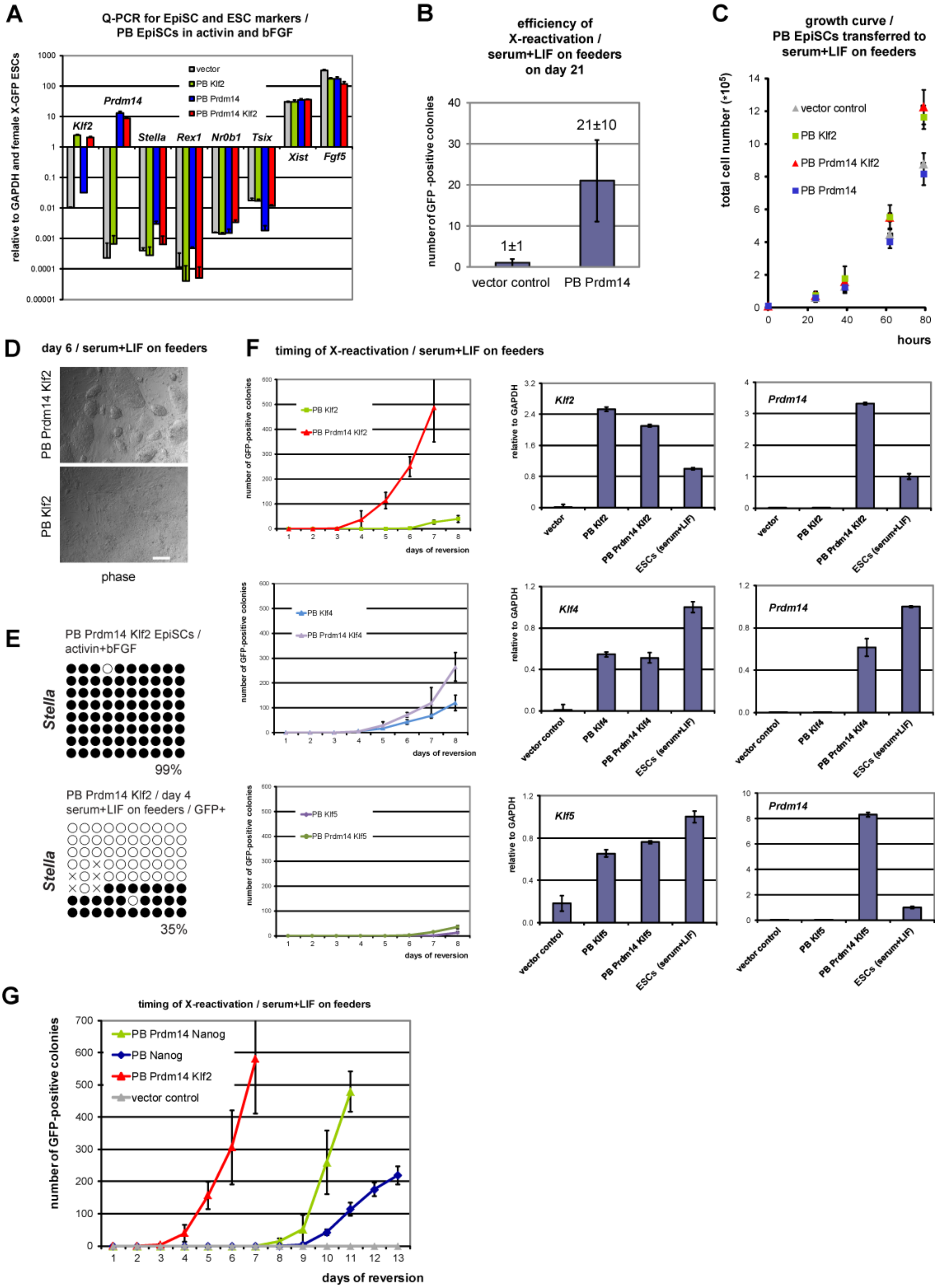

A
Q-PCR for EpiSC and ESC markers / PB EpiSCs in activin and bFGF
relative to GAPDH and female X-GFP ESCs
vector
PB Klf2
PB Prdm14
PB Prdm14 Klf2
Klf2
Prdm14
Stella
Rex1
Nr0b1
Tsix
Xist
Fgf5
B
efficiency of X-reactivation / serum+LIF on feeders on day 21
number of GFP-positive colonies
1±1
21±10
vector control
PB Prdm14
C
growth curve / PB EpiSCs transferred to serum+LIF on feeders
total cell number (*10^5)
vector control
PB Klf2
PB Prdm14 Klf2
PB Prdm14
hours
D
day 6 / serum+LIF on feeders
PB Prdm14 Klf2
PB Klf2
phase
E
PB Prdm14 Klf2 EpiSCs / activin+bFGF
Stella
99%
PB Prdm14 Klf2 / day 4 serum+LIF on feeders / GFP+
Stella
35%
F
timing of X-reactivation / serum+LIF on feeders
number of GFP-positive colonies
PB Klf2
PB Prdm14 Klf2
days of reversion
relative to GAPDH
Klf2
Prdm14
vector
PB Klf2
PB Prdm14 Klf2
ESCs (serum+LIF)
PB Klf4
PB Prdm14 Klf4
Klf4
vector control
PB Klf5
PB Prdm14 Klf5
Klf5
G
timing of X-reactivation / serum+LIF on feeders
number of GFP-positive colonies
PB Prdm14 Nanog
PB Nanog
PB Prdm14 Klf2
vector control
days of reversion

Figure S2. X-Reactivation Dynamics upon Overexpression of Prdm14 and Klf Proteins, Related to Figure 3

(A) Q-PCR analysis of selected EpiSC and ESC marker genes in Xi^{GFP} EpiSCs over-expressing *Klf2, Prdm14, Prdm14* and *Klf2,* or vector control cultured in activin and bFGF on fibronectin relative to GAPDH and female X-GFP ESCs. Error bars are mean±s.d. (n=2).
(B) Quantification of the efficiency of X-chromosome reactivation in Xi^{GFP} EpiSCs overexpressing *Prdm14* compared to vector control on day 21 after transfer to serum and LIF on feeder cells. The cells were passaged twice and the number of GFP-positive colonies was counted after 21 days. Data are shown as mean±s.d. of 3 biological replicates from 50,000 plated cells/6-well.
(C) Growth curve of Xi^{GFP} EpiSCs overexpressing *Klf2*, *Prdm14*, *Prdm14* and *Klf2,* or vector control after transfer to serum and LIF on feeder cells (10,000 plated cells/6-well).
(D) Phase contrast images of Xi^{GFP} EpiSCs overexpressing *Klf2±Prdm14* on day 6 after transfer to serum and LIF on feeder cells. Scale bar, 100 µm.
(E) Bisulfite sequencing analysis of DNA methylation on the *Stella* locus in Xi^{GFP} EpiSCs overexpressing *Prdm14 and Klf2* cultured in activin and bFGF and after transfer to serum and LIF on feeder cells and sorting of GFP-positive cells after 4 days. CpG dinucleotides are shown as open (unmethylated) or filled circles (methylated) with crosses representing mutated or missing CpGs. The numbers show the percentage of methylated CpGs.
(F) Quantification of the timing and efficiency of X-chromosome reactivation upon transfer of Xi^{GFP} EpiSCs overexpressing *Klf2±Prdm14*, *Klf4±Prdm14* and *Klf5±Prdm14* to serum and LIF on feeder cells. The number of GFP-positive colonies was counted every day. Data are shown as mean±s.d. of 3 biological replicates from 30,000 plated cells/6-well. In addition, *Klf2*, *Klf4* and *Klf5* expression levels are shown in stable EpiSC lines as analysed by Q-PCR. Data are shown relative to GAPDH and error bars are mean±s.d. (n=2).
(G) Quantification of the timing and efficiency of X-chromosome reactivation upon transfer of Xi^{GFP} EpiSC lines overexpressing *Nanog*, *Nanog* and *Prdm14*, *Klf2* and *Prdm14,* or vector control to serum and LIF on feeder cells. The number of GFP-positive colonies was counted every day. Data are shown as mean±s.d. of 3 biological replicates from 50,000 plated cells/6-well.

Figure S3

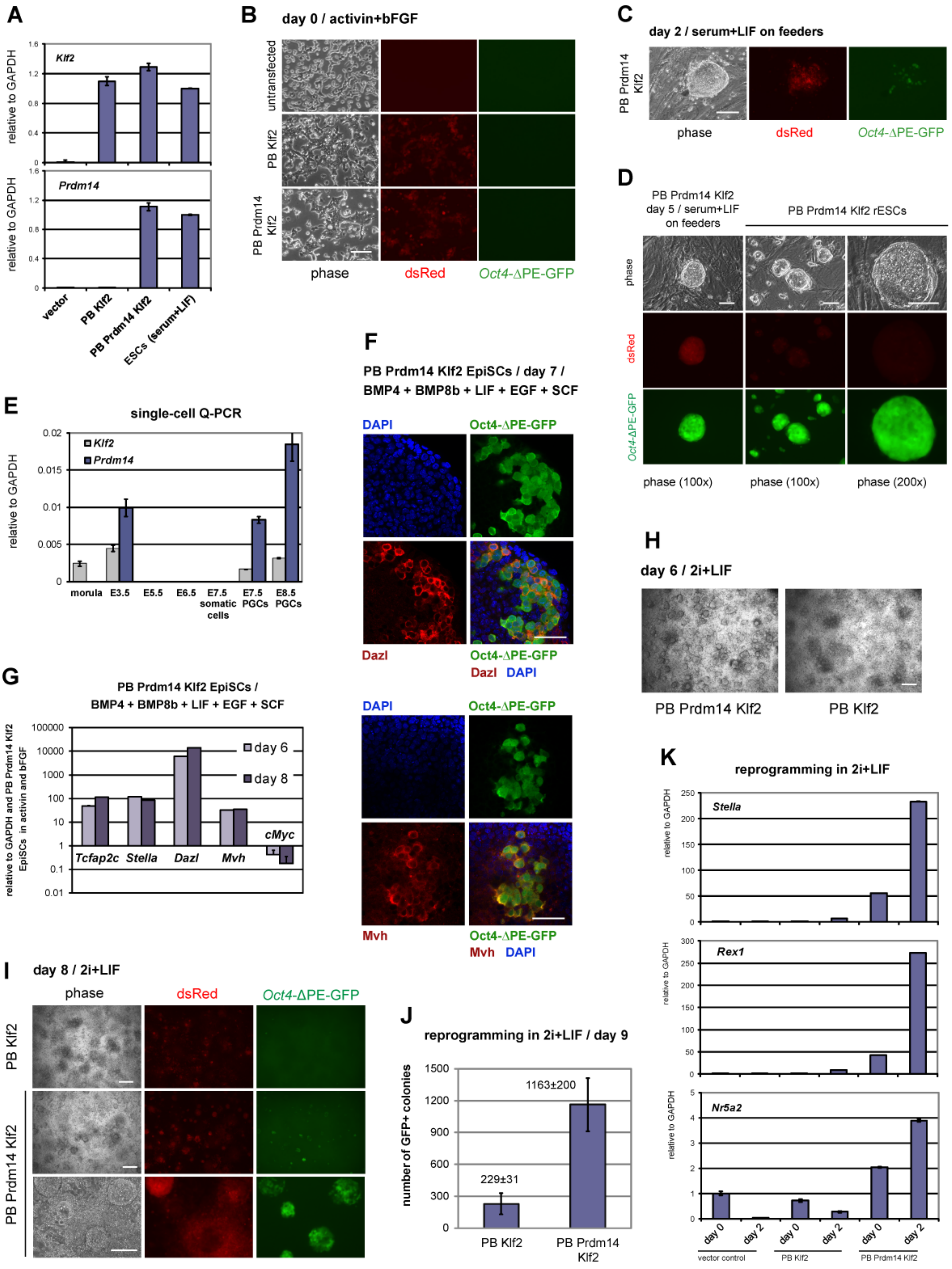

A
Klf2
Prdm14
relative to GAPDH
vector
PB Klf2
PB Prdm14 Klf2
ESCs (serum+LIF)
B
day 0 / activin+bFGF
untransfected
PB Klf2
PB Prdm14 Klf2
phase
dsRed
Oct4-ΔPE-GFP
C
day 2 / serum+LIF on feeders
PB Prdm14 Klf2
phase
dsRed
Oct4-ΔPE-GFP
D
PB Prdm14 Klf2 day 5 / serum+LIF on feeders
PB Prdm14 Klf2 rESCs
phase
dsRed
Oct4-ΔPE-GFP
phase (100x)
phase (100x)
phase (200x)
E
single-cell Q-PCR
Klf2
Prdm14
relative to GAPDH
morula
E3.5
E5.5
E6.5
E7.5 somatic cells
E7.5 PGCs
E8.5 PGCs
F
PB Prdm14 Klf2 EpiSCs / day 7 / BMP4 + BMP8b + LIF + EGF + SCF
DAPI
Oct4-ΔPE-GFP
Dazl
Oct4-ΔPE-GFP Dazl DAPI
DAPI
Oct4-ΔPE-GFP
Mvh
Oct4-ΔPE-GFP Mvh DAPI
G
PB Prdm14 Klf2 EpiSCs / BMP4 + BMP8b + LIF + EGF + SCF
relative to GAPDH and PB Prdm14 Klf2 EpiSCs in activin and bFGF
day 6
day 8
Tcfap2c
Stella
Dazl
Mvh
cMyc
H
day 6 / 2i+LIF
PB Prdm14 Klf2
PB Klf2
I
day 8 / 2i+LIF
phase
dsRed
Oct4-ΔPE-GFP
PB Klf2
PB Prdm14 Klf2
J
reprogramming in 2i+LIF / day 9
number of GFP+ colonies
1163±200
229±31
PB Klf2
PB Prdm14 Klf2
K
reprogramming in 2i+LIF
Stella
Rex1
Nr5a2
relative to GAPDH
day 0
day 2
vector control
PB Klf2
PB Prdm14 Klf2

Figure S3. Epigenetic Reprogramming and Induction of PGC-like Cells upon Overexpression of Prdm14 and Klf2 in EpiSCs, Related to Figure 3

(A) Q-PCR analysis of *Prdm14* and *Klf2* transgene expression in *Oct4*-ΔPE-GFP EpiSCs with *Klf2±Prdm14* or vector control relative to GAPDH. Error bars are mean±s.d. (n=2).
(B) Phase contrast and fluorescence images of *Oct4*-ΔPE-GFP EpiSCs with *Klf2±Prdm14* or untransfected cells cultured in activin and bFGF on fibronectin. Scale bar, 100 µm.
(C) Phase contrast and fluorescence images of *Oct4*-ΔPE-GFP EpiSCs overexpressing *Prdm14* and *Klf2* on day 2 upon transfer to serum and LIF on feeder cells, showing the appearance of GFP-positive cells in a mosaic pattern. Scale bar, 100 µm.
(D) Phase contrast and fluorescence images of a reprogrammed colony of *Oct4*-ΔPE-GFP EpiSCs with *Prdm14* and *Klf2* on day 5 after transfer to serum and LIF on feeder cells. The colony was picked and expanded as rESC line with homogeneous *Oct4*-ΔPE-GFP reporter expression. Scale bars, 100 µm.
(E) Single-cell Q-PCR for *Klf2* and *Prdm14* in morula stage, E3.5 blastocyst, E5.5 and E6.5 post-implantation epiblast cells, in primordial germ cells of E7.5 and E8.5 embryos and in somatic cells of E7.5 embryos. Data are shown relative to GAPDH and error bars are mean±s.d. (n=2).
(F) Immunostaining for GFP, Dazl and mouse Vasa homologue (Mvh) in Oct4-ΔPE-GFP EpiSCs with *Prdm14* and *Klf2* cultured in suspension with BMP4 (500 ng/ml), BMP8b, LIF, EGF and SCF (Ohinata et al., 2009; Hayashi et al., 2011) for 6 days. Nuclei were stained with DAPI. Scale bars, 50 µm.
(G) Q-PCR analysis for *Tcfap2c, Stella, Dazl, Mvh* and *cMyc* expression in Oct4-ΔPE-GFP EpiSCs with *Prdm14* and *Klf2* cultured in suspension with BMP4 (500 ng/ml), BMP8b, LIF, EGF and SCF (Ohinata et al., 2009; Hayashi et al., 2011) for 6 and 8 days. Results are shown relative to GAPDH and EpiSCs with *Prdm14* and *Klf2* cultured in activin and bFGF. Error bars are mean±s.d. (n=2).
(H) Phase contrast images of *Oct4*-ΔPE-GFP EpiSCs overexpressing *Klf2±Prdm14* on day 6 after transfer to 2i and LIF without feeders (60,000 plated cells/6-well). Scale bar, 100 µm.
(I) Phase contrast and fluorescence images of *Oct4*-ΔPE-GFP EpiSCs with *Prdm14* and *Klf2* compared to *Klf2* alone on day 8 after transfer to 2i and LIF without feeders (60,000 plated cells/6-well). Scale bars, 100 µm.
(J) Quantification of the efficiency of *Oct4* distal enhancer activation in *Oct4*-ΔPE-GFP EpiSCs with *Prdm14* and *Klf2* compared to *Klf2* alone on day 9 after transfer to 2i and LIF without feeders. The number of GFP-positive colony patches was counted from 20,000 plated cells/6-well. Data are shown as mean±s.d. of 3 biological replicates.
(K) Q-PCR analysis of *Stella*, *Rex1* and *Nr5a2* expression in *Oct4*-ΔPE-GFP EpiSCs overexpressing *Klf2±Prdm14* or vector control in activin and bFGF (day 0) and after transfer to 2i and LIF (day 2). Data are shown relative to GAPDH and error bars are mean±s.d. (n=2).

Figure S4

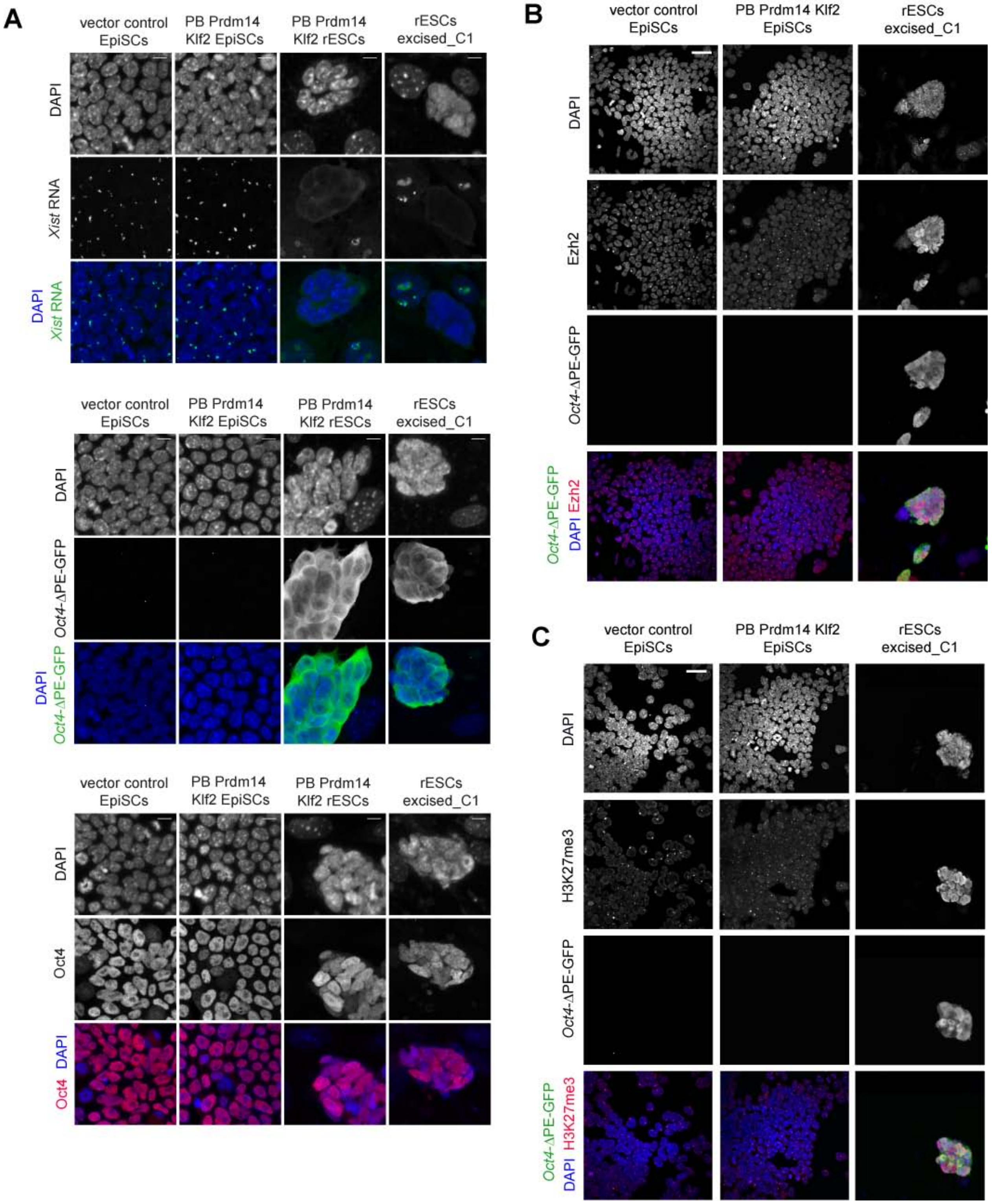

A
vector control EpiSCs
PB Prdm14 Klf2 EpiSCs
PB Prdm14 Klf2 rESCs
rESCs excised_C1
DAPI
Xist RNA
DAPI Xist RNA
Oct4-ΔPE-GFP
DAPI Oct4-ΔPE-GFP
Oct4
Oct4 DAPI
B
vector control EpiSCs
PB Prdm14 Klf2 EpiSCs
rESCs excised_C1
DAPI
Ezh2
Oct4-ΔPE-GFP
Oct4-ΔPE-GFP Ezh2 DAPI
C
vector control EpiSCs
PB Prdm14 Klf2 EpiSCs
rESCs excised_C1
DAPI
H3K27me3
Oct4-ΔPE-GFP
Oct4-ΔPE-GFP H3K27me3 DAPI

Figure S4. Characterisation of rESCs Generated from EpiSCs with Prdm14 and Klf2, Related to Figure 4

(A) RNA-FISH for *Xist* and side-by-side immunostaining for GFP and Oct4 in *Oct4*-ΔPE-GFP EpiSCs overexpressing *Prdm14* and *Klf2* or vector control, in *Oct4*-ΔPE-GFP rESCs with *Prdm14* and *Klf2* transgenes and in *Oct4*-ΔPE-GFP rESC clone C1 with excised transgenes. Nuclei were counterstained with DAPI. Images are projected Z-sections. Scale bars: 10 µm.
(B) Double immunostaining for Ezh2 and GFP in *Oct4*-ΔPE-GFP EpiSCs overexpressing *Prdm14* and *Klf2* or vector control. Nuclei were stained with DAPI. Scale bar, 30 µm.
(C) Double immunostaining for H3K27me3 and GFP in *Oct4*-ΔPE-GFP EpiSCs overexpressing *Prdm14+Klf2* or vector control. Nuclei were stained with DAPI. Scale bar, 25 µm.

Figure S5

A

Q-PCR validation of Illumina microarray

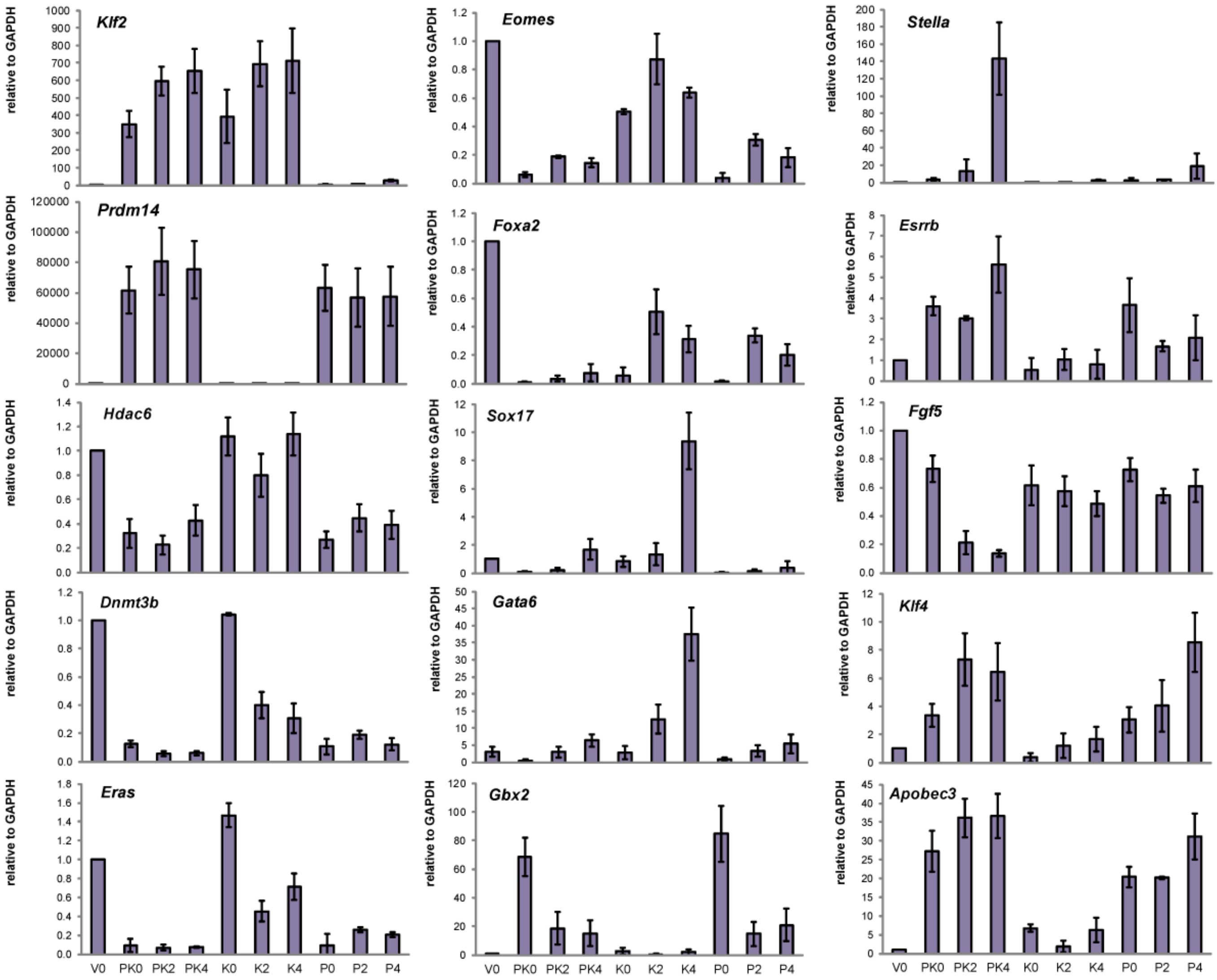

B

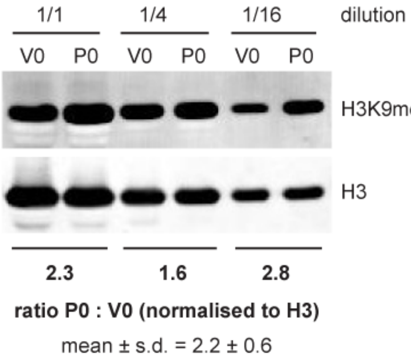

C

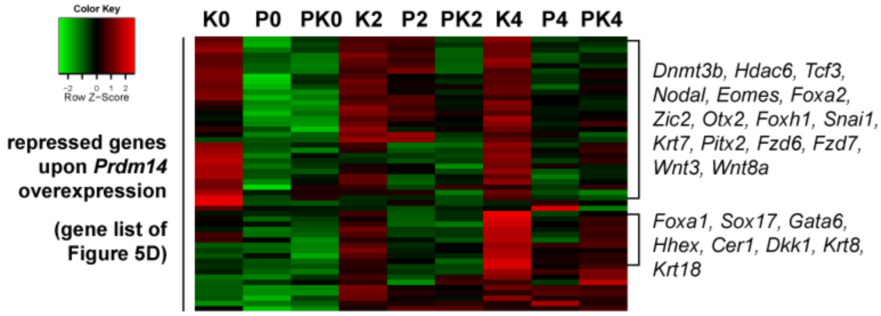

Figure S5. Time-Course Microarray Analysis and Q-PCR Validation, Related to Figure 5

(A) Q-PCR analysis for *Prdm14* and *Klf2* transgene expression and selected genes to validate Illumina microarray gene expression changes. Data are shown relative to GAPDH and as mean±s.d. of 2 independent biological duplicates.
(B) Western blot for H3K9me2 levels in Xi^{GFP} EpiSCs overexpressing *Prdm14* (P0) compared to vector control (V0). H3K9me2 band intensity of P0 versus V0 was quantified using Odyssey and is shown relative to the intensity of the H3 bands.
(C) Heatmap of selected genes that are repressed upon *Prdm14* overexpression in EpiSCs on day 0 compared to vector control (FDR<0.005). The dynamics of these genes is shown as a time-course of day 0 / day 2 / day 4.

Figure S6

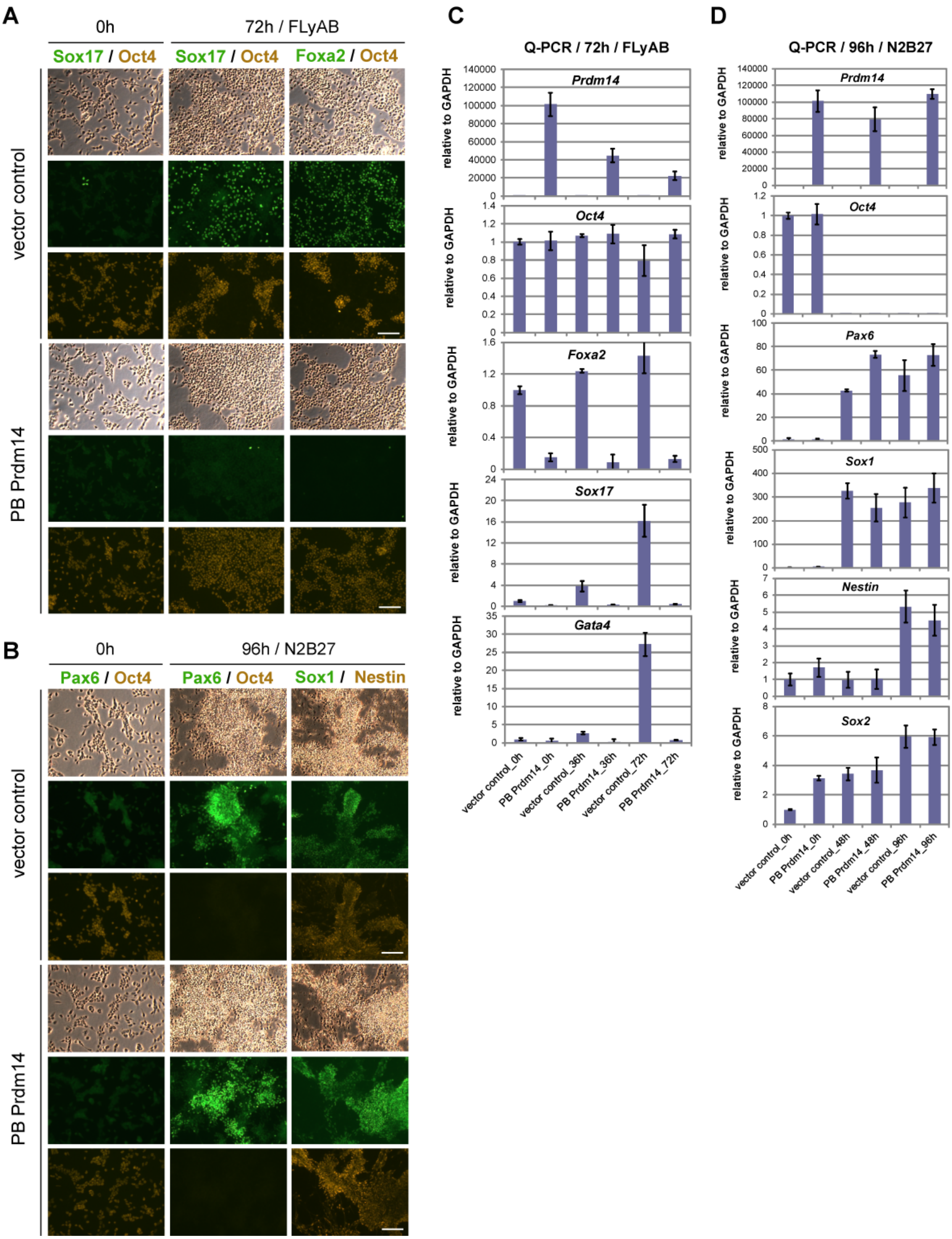
A
0h
72h / FLyAB
Sox17 / Oct4
Sox17 / Oct4
Foxa2 / Oct4
vector control
PB Prdm14
B
0h
96h / N2B27
Pax6 / Oct4
Pax6 / Oct4
Sox1 / Nestin
vector control
PB Prdm14
C
Q-PCR / 72h / FLyAB
relative to GAPDH
Prdm14
Oct4
Foxa2
Sox17
Gata4
vector control_0h
PB Prdm14_0h
vector control_36h
PB Prdm14_36h
vector control_72h
PB Prdm14_72h
D
Q-PCR / 96h / N2B27
relative to GAPDH
Prdm14
Oct4
Pax6
Sox1
Nestin
Sox2
vector control_0h
PB Prdm14_0h
vector control_48h
PB Prdm14_48h
vector control_96h
PB Prdm14_96h

Figure S6. Prdm14 Inhibits EpiSC Differentiation into Endoderm, but not Neuroectoderm, Related to Figure 5

(A) Phase contrast images and immunostaining for Sox17, Foxa2 and Oct4 in EpiSCs overexpressing *Prdm14* or vector control in activin and bFGF (0h) and after 72 hours in “FLyAB” conditions (Touboul et al., 2010; 20 ng/ml bFGF, 10 µM LY294002, 100 ng/ml activin, 10 ng/ml BMP4). Scale bars, 100 µm.
(B) Phase contrast images and immunostaining for Pax6 and Oct4 or Sox1 and Nestin in EpiSCs overexpressing *Prdm14* or vector control in activin and bFGF (0h) and after 96 hours in N2B27 basal medium (Ying et al., 2003). Scale bars, 100 µm.
(C) Q-PCR analysis for *Prdm14, Oct4* and endoderm markers *Foxa2, Sox17* and *Gata4* in EpiSCs overexpressing *Prdm14* or vector control in activin and bFGF (0h) and after 36 and 72 hours in “FLyAB” conditions (Touboul et al., 2010; 20 ng/ml bFGF, 10 µM LY294002, 100 ng/ml activin, 10 ng/ml BMP4). Data are shown relative to GAPDH and as mean±s.d. of 2 independent biological replicates.
(D) Q-PCR analysis for *Prdm14, Oct4* and neuroectodermal markers *Pax6, Sox1, Nestin* and *Sox2* in EpiSCs overexpressing *Prdm14* or vector control in activin and bFGF (0h) and after 48 and 96 hours in N2B27 basal medium (Ying et al., 2003). Data are shown relative to GAPDH and as mean±s.d. of 2 independent biological replicates.

Figure S7

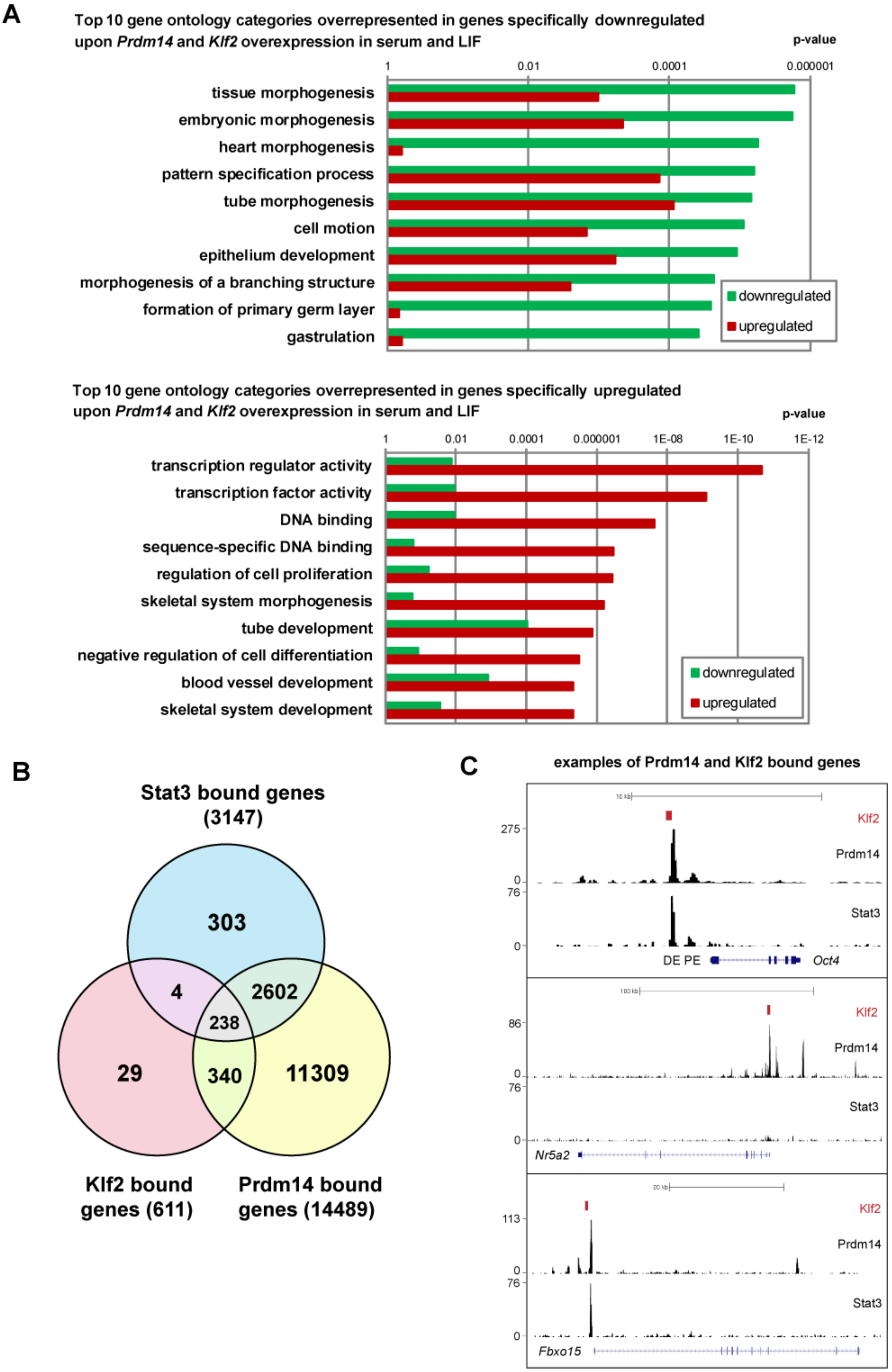
A
Top 10 gene ontology categories overrepresented in genes specifically downregulated upon Prdm14 and Klf2 overexpression in serum and LIF
p-value
1
0.01
0.0001
0.000001
tissue morphogenesis
embryonic morphogenesis
heart morphogenesis
pattern specification process
tube morphogenesis
cell motion
epithelium development
morphogenesis of a branching structure
formation of primary germ layer
gastrulation
downregulated
upregulated
Top 10 gene ontology categories overrepresented in genes specifically upregulated upon Prdm14 and Klf2 overexpression in serum and LIF
p-value
1
0.01
0.0001
0.000001
1E-08
1E-10
1E-12
transcription regulator activity
transcription factor activity
DNA binding
sequence-specific DNA binding
regulation of cell proliferation
skeletal system morphogenesis
tube development
negative regulation of cell differentiation
blood vessel development
skeletal system development
downregulated
upregulated
B
Stat3 bound genes (3147)
303
4
2602
238
29
340
11309
Klf2 bound genes (611)
Prdm14 bound genes (14489)
C
examples of Prdm14 and Klf2 bound genes
275
0
76
0
Klf2
Prdm14
Stat3
DE PE
Oct4
86
0
76
0
Klf2
Prdm14
Stat3
Nr5a2
113
0
76
0
Klf2
Prdm14
Stat3
Fbxo15

Figure S7. Gene Ontology Analysis and Reanalysis of Prdm14, Stat3 and Klf2 Binding Data, Related to Figure 6

(A) Top 10 DAVID gene ontology categories overrepresented in genes specifically down- or upregulated upon *Prdm14* and *Klf2* overexpression compared to the factors overexpressed individually upon transfer to serum and LIF on feeder cells (FDR<0.005).
(B) Venn diagram showing the extent of overlap between published Klf2 ChIP-on-chip targets (611 identified targets; Jiang et al., 2008) with Prdm14 ChIP-Seq targets (Ma et al., 2011) and Stat3 ChIP-Seq targets (Chen et al., 2008).
(C) UCSC Genome Browser visualisation of binding sites for Prdm14, Stat3 and Klf2 (red bar) on *Oct4 DE*, *Nr5a2* and *Fbxo15* loci that are bound by all 3 factors, as identified by Klf2 ChIP-on-chip (Jiang et al., 2008), Prdm14 ChIP-Seq (Ma et al., 2011) and Stat3 ChIP-Seq analysis (Chen et al., 2008).

SUPPLEMENTAL EXPERIMENTAL PROCEDURES

EpiSC Derivation and Culture

Animal studies were authorised by a UK Home Office Project License and carried out in a Home Office designated facility. Noon on the day of vaginal plug detection was designated as E0.5. EpiSCs were derived from E6.5 epiblasts of 129/SvEv females crossed with X-GFP transgenic male mice (Hadjantonakis et al., 2001; mixed background of 129 and ICR strains) or with *Oct4*-ΔPE-GFP transgenic male mice (Yeom et al., 1996; mixed background of MF1, 129/SvEv and C57BL/6J strains). Female Xi^{GFP} EpiSCs were established from $XmXp^{GFP}$ EpiSCs by 3 consecutive rounds of flow cytometric sorting of the GFP-negative cell population. Blimp1-knockout EpiSCs were generated by injection of Blimp1-null ESCs (129 background) into wildtype tetraploid host blastocysts generated by electrofusion, subsequent transfer to E2.5 pseudopregnant recipients and derivation of EpiSCs at E6.5. Dissected epiblasts were placed in trypsin/pancreatin solution for 8 minutes at room temperature and pulled through a fine glass needle to remove the visceral endoderm. The epiblasts were cultured in chemically defined medium (Brons et al., 2007) supplemented with 20% knockout serum replacement (Gibco), recombinant human activin A (20 ng/ml; Peprotech) and bFGF (12 ng/ml; Invitrogen) on mitomycin C-treated MEFs. EpiSCs were passaged every 2 days at a split ratio of 1:2 to 1:4 by incubation in Collagenase Type IV (Invitrogen) for 6 minutes at room temperature and by manual dissociation into small clumps by careful pipetting. After 4-8 initial passages on mitomycin C-treated MEFs, EpiSCs were cultured feeder-free on fibronectin (Millipore) coated dishes in N2B27 medium: DMEM/F12 (Gibco) with N2 and B27 supplement (Invitrogen), 2 mM L-glutamine (Gibco), 0.1 mM MEM non-

essential amino acids (Gibco), 0.1 mM 2-mercaptoethanol (Gibco), 50 µg/ml bovine serum albumin (BSA; Sigma), 100 U/ml penicillin and 100 µg/ml streptomycin (Gibco), supplemented with recombinant human activin A (20 ng/ml; Peprotech) and bFGF (12 ng/ml; Invitrogen). EpiSCs were passaged using Accutase (PAA) at a split ratio of 1:2 to 1:4 every 1-2 days.

Generation of EpiSCs with Stable Overexpression of Candidate Factors

1×10^6 EpiSCs were transfected with 0.7 µg of PB-*GOI1-dsRed-IRES-blast* plasmid, 0.7 µg of PB-*GOI2-hygro* plasmid and 2.6 µg *pBase* (Wang et al., 2008) using Lipofectamine 2000 (Invitrogen). Transfectants were selected in hygromycin (175 µg/ml; Invitrogen) and blasticidin (5 µg/ml; Invitrogen) for 5-7 days. For overexpression of 3 factors, 1×10^6 hygromycin-blasticidin double resistant EpiSCs containing two transgenes were transfected again with 1.4 µg of PB-*GOI3-zeo* plasmid and 2.6 µg *pBase*. Transfectants were selected in zeocin (100 µg/ml; Invitrogen) for another 5 days.

Mouse ESC and MEF Culture

Mouse ESCs and rESCs were grown on mitomycin C-treated MEFs in DMEM/F12 medium (Gibco) supplemented with 20% fetal bovine serum (Gibco), 2 mM L-glutamine (Gibco), 0.1 mM MEM non-essential amino acids (Gibco), 100 U/ml penicillin and 100 µg/ml streptomycin (Gibco), 1 mM sodium pyruvate (Sigma), 1.2 mg/ml sodium bicarbonate (Sigma), 0.05 mM 2-mercaptoethanol (Gibco) and recombinant mouse LIF (1000 U/ml; ESGRO; Chemicon). Alternatively, (r)ESCs were maintained feeder-free in 2i/LIF conditions (Ying et al., 2008) on gelatin: N2B27

medium (Stem Cell Sciences) supplemented with LIF (1000 U/ml; ESGRO; Chemicon), the Mek inhibitor PD0325901 (1 µM; Stemgent) and the Gsk3 inhibitor CHIRON99021 (3 µM; Stemgent). The (r)ESCs were passaged by trypsin dissociation at a split ratio of 1:5 to 1:10 every 2-3 days.

MEFs were grown on gelatin coated dishes in DMEM medium (Gibco) supplemented with 10% fetal bovine serum (Gibco), 2 mM L-glutamine (Gibco), 100 U/ml penicillin and 100 µg/ml streptomycin (Gibco).

EpiSC Reprogramming Assays in 2i/LIF

Pooled stable transfectants were plated in N2B27 medium supplemented with human activin A (20 ng/ml; Peprotech) and bFGF (12 ng/ml; Invitrogen) at a density of 20,000/60,000 cells per well of a 6-well tissue culture plate coated with fibronectin. After 24 hours, the medium was replaced with N2B27 medium supplemented with LIF (1000 U/ml), the Mek inhibitor PD0325901 (1 µM; Stemgent) and the Gsk3 inhibitor CHIRON99021 (3 µM; Stemgent) and subsequently refreshed every day.

Unless otherwise indicated, the number of GFP-positive colony patches per well of a 6-well plate was counted every day using an Olympus IX71 inverted microscope and results are shown as mean±standard deviation (s.d.) of 3 independent experiments.

Growth Curves

Pooled stable transfectants were plated at a density of 10,000 and 50,000 cells per well of a 6-well tissue culture plate in standard ESC medium containing LIF (1000 U/ml; ESGRO; Chemicon) and 20% fetal bovine serum (Gibco) on mitomycin C-

treated MEFs, that had been pre-plated 5 days earlier at 37˚C and 5% CO_2. The medium was first replaced after 48 hours. The cells were counted in 3 independently seeded wells of the 2 different seeding densities using a hemocytometer every day. The number of feeder cells was substracted by counting the number of feeder cells, that had been incubated in standard ESC medium (LIF, 20% fetal bovine serum) in a separate well, every day. Results are shown as mean±s.d. of 3 independent experiments.

Transgene Excision

1×10^6 rESCs were transiently transfected with 4 µg of Cre expression plasmid using Lipofectamine 2000 (Invitrogen). Three days after transfection dsRed-negative cells were sorted on a MoFlo high-speed cell sorter (Dako Cytomation) and plated at clonal density in standard ESC medium containing LIF (1000 U/ml; ESGRO; Chemicon) and 20% fetal bovine serum (Gibco) on mitomycin C-treated MEFs. Clones were picked manually under the microscope, expanded and tested for transgene excision by genomic PCR (see Table S5 for primer sequences).

8-Cell Injections and Chimera Production

Following transgene excision, 5-6 rESCs were injected into E2.5 C57BL/6 morula stage embryos. Embryos were cultured under oil in M16 medium (Sigma) supplemented with 10% fetal bovine serum (Gibco) or in standard ESC medium containing LIF (1000 U/ml; ESGRO; Chemicon) and 10% fetal bovine serum (Gibco) at 37˚C and 5% CO_2 and inspected for inner cell mass integration after 1-2 days. Chimeras were generated by microinjecting 15-20 rESCs into E3.5 C57BL/6

blastocysts and chimerism was assessed by agouti coat colour. Germ line contribution was verified by inspecting E13.5 C57BL/6 male and female genital ridges for *Oct4*-ΔPE-GFP-positive cells.

Flow Cytometry

EpiSCs were dissociated using Accutase (PAA) and incubated with a phycoerythrin-conjugated mouse IgM anti-SSEA1 antibody (R&D Systems, FAB2155P, 10 µl antibody/10^6 cells) in 0.5% BSA in PBS for 30 minutes at 4°C in the dark and washed twice with 0.5% BSA in PBS. The filtered cell suspension was sorted using a FACSAria cell sorter (BD Biosciences) or a MoFlo high-speed cell sorter (Dako Cytomation).

EpiSC Differentiation Assays

For EpiSC differentiation into endoderm, we used a protocol for endoderm differentiation of human ESCs in monolayer culture (Touboul et al., 2010). Briefly, EpiSCs were seeded on fibronectin (250,000 cells/6-well) and the medium was changed to N2B27 medium (Stem Cell Sciences) containing activin A (100 ng/ml; produced in-house), bFGF (20 ng/ml; produced in-house), BMP4 (10 ng/ml; produced in-house) and the PI3K inhibitor LY294002 (10 µM, Promega, Cat. No. V1201) the following day (“FLyAB” conditions). The medium was changed every day and the cells were analysed after 0 and 72 hours by immunofluorescence and after 0, 36 and 72 hours by Q-PCR.

For EpiSC differentiation into neuroectoderm, we followed a protocol for neuronal differentiation of mouse ESCs in monolayer culture (Ying et al., 2003). Briefly,

EpiSCs were seeded on laminin (250,000 cells/6-well) and the medium was changed to N2B27 basal medium (Stem Cell Sciences) the following day. The medium was changed every day and the cells were analysed after 0 and 96 hours by immunofluorescence and after 0, 48 and 96 hours by Q-PCR.

Phase and fluorescence images were acquired on an Olympus IX71 inverted microscope (10× objective).

Induction of PGC-like Cells from EpiSCs

Induction of PGC-like cells from EpiSCs in suspension culture was performed as described previously (Ohinata et al., 2009; Hayashi et al., 2011). Briefly, 1000 EpiSCs were seeded per well of a low-cell-binding 96-well plate (Nunc) in 200 µl of GMEM (Gibco) with 15% KSR, 2 mM L-glutamine, 0.1 mM NEAA, 1 mM sodium pyruvate, 0.1 mM 2-mercaptoethanol, 100 U/ml penicillin and 100 µg/ml streptomycin (Gibco), supplemented with recombinant BMP4 (500 ng/ml; produced in-house), LIF (1000 U/ml; ESGRO; Chemicon), SCF (100 ng/ml; R&D Systems), BMP8b (500 ng/ml; R&D Systems) and EGF (50 ng/ml; R&D Systems). Aggregates were harvested after 6, 7 or 8 days and analysed by immunofluorescence (Olympus FV1000 Upright confocal microscope) or by Q-PCR.

Immunofluorescence and *Xist* RNA Fluorescence in Situ Hybridisation (RNA-FISH)

EpiSCs were grown on fibronectin coated coverslips, washed once in PBS, fixed with 4% paraformaldehyde for 15 minutes at room temperature and permeabilised with blocking solution (PBS with 0.1% Triton X-100 and 0.1% BSA) for 5 minutes. Cells

were incubated with first antibody diluted in blocking solution at 4˚C overnight: rabbit anti-Nanog (Cosmo Bio, REC-RCAB0001P, 1:500), mouse anti-Sox2 (R&D Systems, MAB2018, 1:50), mouse anti-Oct4 (BD Biosciences, 611203, 1:200), rabbit anti-H3K27me3 (Upstate/Millipore, 07-449, 1:500), mouse anti-Ezh2 (Cell Signaling Technology, 3147, clone AC22, 1:50), rat anti-GFP (Nacalai Tesque, 04404-26, 1:500), rabbit anti-Stella (Dppa3/PGC7, kind gift by Toru Nakano, 1:5000), goat anti-Sox17 (R&D Systems, AF1924, 1:100), rabbit anti-Foxa2 (Cell Signaling Technology, 3143S, 1:200), rabbit anti-Pax6 (Covance, PRB-278P, 1:300), rabbit anti-Sox1 (Cell Signaling Technology, 4194S, 1:200), mouse anti-Nestin (BD Biosciences, 556309, 1:200), rabbit Dazl (Abcam, ab34139, 1:500), rabbit Mvh (Abcam, ab13840, 1:500). After three 10 minute washes in blocking solution, the samples were incubated with Alexa Fluor 488/568/647 donkey anti-mouse/rabbit/rat/goat secondary antibodies (Invitrogen, 1:500) diluted in blocking solution at 4˚C overnight in the dark. Cells were washed in blocking solution 3 times for 10 minutes, mounted in Vectashield with DAPI (Vector laboratories) and analysed with a BioRad Radiance 2100 confocal microscope (40× oil objective) or an Olympus FV1000 Upright confocal microscope (60× oil objective).

For ubiquitinated H2A staining, EpiSCs were incubated in ice-cold cytoskeletal buffer (100 mM NaCl, 300 mM sucrose, 3 mM $MgCl_2$, 10 mM PIPES/pH 6.8) for 30 seconds, in cytoskeletal buffer with 0.5% Triton X-100 for 30 seconds and in cytoskeletal buffer for 30 seconds. Cells were fixed with 4% paraformaldehyde for 15 minutes at room temperature, washed in 0.2% Tween-20/PBS three times for 5 minutes and incubated in blocking solution (PBS with 5% goat serum, 0.2% Tween-20 and 0.2% gelatin) for 30 minutes at 37˚C. Samples were incubated with mouse IgM anti-ubiquityl-histone H2A (Upstate/Millipore, 05-678, clone E6C5, 1:50) in

blocking solution for 1 hour at 37˚C. After three 10 minute washes in PBS with 0.2% Tween, the cells were incubated with Alexa Fluor 488 goat anti-mouse IgM secondary antibody (Invitrogen, 1:500) in blocking solution for 30 minutes at 37˚C in the dark. The cells were washed three times for 10 minutes in PBS with 0.2% Tween, mounted and analysed as described above.

Xist RNA-FISH was performed as described previously (Pasque et al., 2011). Samples were analysed on a Zeiss LSM 510 Meta confocal microscope (60× oil objective). Images were processed using the ImageJ despeckle function and Z-sections were projected on a single plane using the ImageJ Z-project standard deviation function.

Quantitative Real-Time PCR (Q-PCR)

Total RNA was extracted using the RNeasy Mini Kit (Qiagen) with a Qiashredder and on-column DNase I (Qiagen) digestion. 500 ng of total RNA were used for cDNA synthesis using Superscript III (Invitrogen) and random hexamer primers (Invitrogen). Q-PCR reactions were set up with Sybr Green JumpStart Taq ReadyMix (Sigma) and 1 µM of forward and reverse gene-specific primers (see Table S5 for primer sequences). Amplification was performed with an ABI Prism 7000 Sequence Detection System (Applied Biosystems) at 95˚C for 10 minutes, 40 cycles of 95˚C for 15 seconds and 60˚C for 1 minute, followed by a melting curve. Mean threshold cycles were determined from two technical replicates unless otherwise stated using the comparative Ct method and expression levels were normalised to GAPDH. Single-cell Q-PCR was performed as described previously (Kurimoto et al., 2007; Tang et al., 2010).

Western Blotting

Cells were lysed in hypotonic lysis buffer (1 mM KCl, 1.5 mM $MgCl_2$, 1 mM DTT, 0.4 mM PMSF, 10 mM Tris-HCl pH 8.0 with Roche protease inhibitor cocktail) for 30 minutes at 4˚C and centrifuged. The pellet was resupended in 0.4 M H_2SO_4 and incubated overnight at 4˚C. After centrifugation 1/3 volume of trichloroacetic acid was added to the supernatant. The samples were incubated for 30 minutes on ice, centrifuged and the pellet was resuspended in dH_20 after 2 washes in acetone. The samples were boiled for 5 minutes at 95˚C in Laemmli sample buffer, separated on a 15% acrylamide/SDS gel and transferred onto a PVDF membrane (Millipore Immobilion-P) using a wet transfer system. The membrane was incubated in blocking solution [5% dried skimmed milk in Tris buffered saline (TBS) pH 8.8 with 0.1% Tween-20] for 30 minutes at room temperature and with primary antibody diluted in blocking solution at 4˚C overnight: rabbit anti-H3K9me2 (Upstate, 07-441, 1:500), rabbit anti-Histone H3 (Abcam, ab1791, 1:5000). The membrane was washed in TBS with 0.1% Tween-20 four times for 5 minutes and incubated with anti-rabbit dye conjugated antibody (LI-COR, 1:20000 diluted in blocking solution with 0.01% SDS) for 1 hour at room temperature. After four 5 minute washes in TBS with 0.1% Tween-20 the membrane was scanned with an Odyssey Imager (LI-COR) and bands were quantified using the Odyssey software version 1.2.

Microarray Analysis and Reanalysis of Published ChIP-Seq Data

EpiSCs were cultured feeder-free on fibronectin in N2B27 medium with human activin A (20 ng/ml; Peprotech) and bFGF (12 ng/ml; Invitrogen) (samples D0) or transferred to standard ESC medium with LIF (1000 U/ml; ESGRO; Chemicon) and

20% fetal bovine serum (Gibco) on mitomycin C-treated MEFs for 2 or 4 days and sorted for dsRed expression to remove feeder cells (samples D2 and D4). RNA was isolated from two biological replicates using the RNeasy Mini Kit (Qiagen) with a Quiashredder and on-column DNase I (Qiagen) digestion. After RNA quality control using the Bioanalyzer, the samples were processed and hybridised to Illumina Mouse WG-6 v2.0 Expression BeadChips by Cambridge Genomic Services, who also performed data quality control.

Raw microarray data have been submitted to the ArrayExpress repository (http://www.ebi.ac.uk/arrayexpress) with the accession number E-MTAB-782.

Raw bead-level data were imported into the R (http://www.r-project.org) statistical programming environment using functionality of the *beadarray* package from the associated *Bioconductor* (http://bioconductor.org) suite of open-source bioinformatics analysis software. Raw probe intensities were background corrected using the RMA algorithm (Irizarry et al., 2003), $\log_2$ transformed and summarised using *Bioconductor:beadarray*. Processed expression profiles were subsequently quantile normalised using *Bioconductor:limma*. For each probe-set, differential expression between groups of two or more profiles was assessed using non-parametric z-values on the $\log_2$ ratios of mean group expressions. The significance p-values obtained were corrected for multiple testing using the false-discovery method (Storey and Tibshirani, 2003). Differential expression was deemed significant at a false discovery rate (FDR) of 0.5% (corrected p-value < 0.005).

Heatmaps of comparative gene expression were generated using the *heatmap.2* function of *Bioconductor:gplots*. In the case wherein a gene was represented by more than one microarray probe-set, the probe-set with highest average expression

across all samples was selected. Gene ontology analysis across selected gene subsets of interest was performed using the DAVID bioinformatics resources as described (Huang da et al., 2009) (http://david.abcc.ncifcrf.gov).

Reanalysis of published Prdm14 ChIP-Seq data (Ma et al., 2011) and Stat3 ChIP-Seq data (Chen et al., 2008) involved alignment of both ChIP and input short-read sequences to the mm9 genome using the *Bowtie* short-read aligner (Langmead et al., 2009). Alignment options were default values except restriction to one genomic location per read ('best' alignment retained) and zero or two mismatches allowed for Prdm14 and Stat3 respectively. Reads that overlapped the UCSC mm9 repeatmask (http://genome.ucsc.edu) were removed. ChIP-bound regions (peaks) were called with the MACS1.4 peak-finder (Zhang et al., 2008) (p<1e-9; FDR 0.2%). The *Bioconductor* packages *biomaRt* and *IRanges* were used to associate peaks with unique *Ensembl61* transcript regions if located within 100 kb upstream and 2 kb downstream of the TSS/TES respectively. Published Klf2 binding locations from ChIP-on-chip data (Jiang et al., 2008) were lifted over from mm5 to mm9 using the *Galaxy* online software suite (http://main.g2.bx.psu.edu).

Bound regions, defined as described above, were matched to corresponding Illumina BeadArray probe-sets using correspondence between associated EntrezGene identifiers. Statistical over-representation of probe-sets with associated ChIP-bound regions among all those deemed differentially regulated was performed using hyper-geometric statistics as implemented in the *R:stats* library.

Chromatin Immunoprecipitation (ChIP)

EpiSCs were cultured feeder-free on fibronectin in N2B27 medium with human activin A (20 ng/ml; Peprotech) and bFGF (12 ng/ml; Invitrogen) or transferred to standard ESC medium (LIF; 20% fetal bovine serum) on mitomycin C-treated MEFs for 2 days and pre-plated for 30 minutes on gelatin to remove feeder cells. ChIP was performed as described (Lee et al., 2006) with minor modifications. The cells were crosslinked in 1% formaldehyde for 10 minutes at room temperature and quenched with 0.125 M glycine for 5 minutes at room temperature. Nuclei were isolated by 3-step lysis in lysis buffer 1 (50 mM Hepes pH 7.5, 140 mM NaCl, 1 mM EDTA, 10% glycerol, 0.5% NP-40, 0.25% Triton X-100 in dH_2O) for 10 minutes at 4˚C, in lysis buffer 2 (10 mM Tris pH 8.0, 200 mM NaCl, 1 mM EDTA, 0.5 mM EGTA in dH_2O) for 10 minutes at room temperature, and in lysis buffer 3 (10 mM Tris pH 8.0, 100 mM NaCl, 1 mM EDTA, 0.5 mM EGTA, 0.1% sodium deoxycholate, 0.5% N-lauroylsarcosine in dH_2O). Chromatin was sonicated to an average fragment length of 500 bp using a tip sonicator (7×20 seconds; 30% amplitude). Samples were immunoprecipitated overnight at 4˚C with 5 µg antibody, that had been preincubated with rabbit protein G Dynabeads (Invitrogen) in PBS with 0.5% BSA for 6 hours at 4˚C: rabbit anti-Klf2 (kind gift by Huck-Hui Ng), rabbit anti-Nanog (Cosmo Bio; REC-RCAB0001P), goat anti-Sox2 (Santa Cruz; sc-17320), goat anti-Oct4 (Santa Cruz; sc-8628), rabbit IgG (Santa Cruz; sc-2027), goat IgG (Santa Cruz; sc-2028). The next day, beads were washed 5 times in RIPA buffer (50 mM Hepes pH 7.6, 0.5 M EDTA, 0.7% sodium deoxycholate, 1% NP-40, 0.5 M lithium chloride in dH_2O) and once with TE buffer (10 mM Tris pH 8.0 with 10 mM EDTA in dH_2O) with 50 mM NaCl. Elution was performed for 15 minutes at 65˚C in elution buffer (50 mM Tris pH 8.0, 10 mM EDTA, 1% SDS in dH_2O) and crosslinking was reversed by incubation

for 6 hours at 65˚C. After RNase A (0.2 µg/µl; Sigma) and proteinase K treatment (0.2 µg/µl; Invitrogen) for 1 hour at 37˚C and 2 hours at 55˚C, respectively, the DNA was extracted twice with phenol:chloroform (Ambion) using phaselock tubes, ethanol precipitated and analysed by Q-PCR (see Table S5 for primer sequences). Data were normalised to input and are displayed as % input. Data are shown as mean±s.d. of 3 independent biological replicates.

Bisulfite Sequencing

For genomic DNA isolation, the cell pellet was incubated in tail extraction buffer (50 mM Tris pH 7.6, 100 mM EDTA pH 8.0, 1% SDS) with proteinase K (200 µg/ml; Invitrogen) for 4 hours at 55˚C and for 10 minutes at 95˚C. The DNA was precipitated using isopropanol, centrifuged for 30 minutes at 4˚C and maximum speed and the DNA pellet was resuspended in dH_2O. Bisulfite conversion was performed on 800 ng of isolated genomic DNA with the EpiTect Bisulfite Kit (Qiagen). The converted DNA was amplified by nested PCR using the primers previously described (Hayashi and Surani, 2009; Lucifero et al., 2002); see Table S5 for primer sequences).

<u>PCR amplification conditions:</u>

Stella: First amplification for 3 minutes at 94˚C, 30 cycles of 30 seconds at 94˚C, 30 seconds at 50˚C and 1 minute at 72˚C, followed by final extension for 3 minutes at 72˚C. Second amplification for 3 minutes at 94˚C, 20 cycles of 30 seconds at 94˚C, 30 seconds at 50˚C and 1 minute at 72˚C, followed by final extension for 3 minutes at 72˚C. The PCR fragment has a size of 340 bp and the sequence includes 10 CpG dinucleotides.

Peg1, Peg3, Snrpn: Two cycles of 4 minutes at 94˚C, 2 minutes at 55˚C and 2 minutes at 72˚C, followed by 35 cycles of 1 minute at 94˚C, 2 minutes at 55˚C and 2 minutes at 72˚C for first amplification. Second amplification for 35 cycles of 1 minute at 94˚C, 2 minutes at 55˚C and 2 minutes at 72˚C. The PCR fragments have a size of 563 bp for *Peg1*, 444 bp for *Peg3* and 420 bp for *Snrpn*. The sequence includes 23, 29 and 16 CpG dinucleotides for *Peg1*, *Peg3* and *Snrpn*, respectively.

The PCR products were cloned into the pGEM-T Easy vector (Promega) and clones were sequenced by Cogenics. Sequences were analysed using the Qantification Tool for Methylation Analysis (QUMA, http://quma.cdb.riken.jp).

Table S5. Sequences of primers used in this study

Primer Name	Forward (5’-3’)	Reverse (5’-3’)
**Genomic PCR Primers**		
PB LTR (Yang et al., 2010)	CCCTAGAAAGATAATCATATTGTGAC	CCCTAGAAAGATAGTCTGCGTAAAAT
Klf2 (transgene: 360bp)	GCCTTCGGTCTTTTCGAGGA	CGCACAAGTGGCACTGAAAG
Prdm14 (transgene: 265bp)	TTGGTGATGTGCCACACTTT	AATATCTGCCCTTGGTGCTG
**Q-PCR Primers (cDNA synthesis: random hexamer primers)**		
Gapdh	CATGGCCTTCCGTGTTCCT	GCGGCACGTCAGATCCA
Oct4 (Pou5f1)	TGGATCCTCGAACCTGGCTA	CCCTCCGCAGAACTCGTATG
Nanog	AATGCTGCTCCGCTCCATAA	TAAAATGCGCATGGCTTTCC
Sox2	GGAGAACCCCAAGATGCACA	GCTTCTCGGTCTCGGACAAA
Klf2	TAAAGGCGCATCTGCGTACA	CGCACAAGTGGCACTGAAAG
Prdm14	GCATCCTGGTTCCCACAGAG	CTGCAGAACACGCCAAAGTG
Stella (Dppa3)	AGCGCCTTTCCCAAGAGAAG	AGGGTCTTTCAGCACCGACA
Rex1 (Zfp42)	GGCCTCTTTTGGTATTCCATGG	CCCATCCCCTTCAATAGCACAT
Nr0B1	TCCAGGCCATCAAGAGTTTC	ATCTGCTGGGTTCTCCACTG
Tsix	TGTCAGGTTTCGGGGACACT	CTCTCCAGCCCAGGAACTGA
Xist	CTCATAGTAGTGGCCGAGTA	TAAGCCCGTTAAGTAGTCCTT
Fgf5	AAACTCCATGCAAGTGCCAAAT	TCTCGGCCTGTCTTTTCAGTTC
Nr5a2	GCGAGGGGGCAGAAATAAGT	GGCTCGAATGAGGGCTTTCT
Klf4	GCACACCTGCGAACTCACAC	GTTTGCGGTAGTGCCTGGTC
Klf5	TTGCTTCCAAACTGGCGATT	AGGTGGGAGAGTTGGCGAAT
T (Brachyury)	GAGACGGCTGTGGTCCAGTT	GGGTGGACGAATTCCAGGAT
Lefty1	TGGACAAGGCTGATGTGGAA	TGGCATGGCTGTGTTGTAGC
GLP (Ehmt1)	GTTCCCCATTACATGCTGCT	TGCATCCAAGTGGTTGTTCT
Uhrf1	TCAAAGAGGACAAGGGCAAC	GATGCACTGGAAAGCCTCTT
Dnmt1	CCATGGCTGACACTAAGCTG	ACCAAACCAAACCAAACCAA
Dnmt3a	GACTCGCGTGCAATAACCTTAG	GGTCACTTTCCCTCACTCTGG
Dnmt3b	CTCGCAAGGTGTGGGCTTTTGTAAC	CTGGGCATCTGTCATCTTTGCACC
Hdac6	TCCTCAGCTGTGTTGACCTG	CATGTCCTCCCCAAACTTGT
Eras	GTAGCTGTGGCTGCTCTGTAG	GATGTCTGTGGTAACTTGGTCG
Eomes	GCGGGGAAAACAAACAAACA	GCCAGCCCTACAACAAATGG
Foxa2	TCTCCGTGTCAGGAGCACAA	AGGCAGGTGCTCCCTTTAGC
Sox17	GAATCCAACCAGCCCACTGA	GAGGTTCACTCCGCAGTCGT
Gata4	TTCCTCTCCCAGGAACATCAAA	GCTGCACAACTGGGCTCTACTT
Gata6	TGCTGGAAATTGCAACAAACC	GTCACGTGGTACAGGCGTCA
Gbx2	GCAGTCGGTTGATTTTGGAG	CGACATGGCTCAGATAGGAT
Esrrb	CACTTGGGGACCAGATGAGC	CGGTACACGATGCCCAAGAT

Apobec3	ACTTTGTGAACCCGAAAAGG	GGGGTCCAAGCTGTAGGTTT
Pax6	CTCGGGGACCACTTCAACAG	GGACGGGAACTGACACTCCA
Sox1	CCTGTGGTTCTGCCTTTTGC	TGAGCACAACCCATCCTCCT
Nestin	GATCGCTCAGATCCTGGAAG	AGGTGTCTGCAAGCGAGAGT
Tcfap2c	TGAAGATGAAGCTGGGCTTT	TCCATTCTCTTCCGGTTCAG
cMyc	GACAGAACTGATGCGCTGGA	CCAGCCAAGGTTGTGAGGTT
Mvh	TTCTTCTGTTCTTCCTCCCAACC	GCAGTGTTGTAACGTCAGCATTTC
**Bisulfite Primers**		
Stella outside	ATTTTGTGATTAGGGTTGGTTTAGAA	CCAAAACATCCTCTTCATCTTTCTTCT
Stella inside	TTTTTGGAATTGGTTGGGATTG	CTTCTAAAAAATTTCAAAATCCTTCATT
Peg1 outside	GATTTGGGATATAAAAGGTTAATGAG	TCATTAAAAACACAAACCTCCTTTAC
Peg1 inside	TTTTAGATTTTGAGGGTTTTAGGTTG	AATCCCTTAAAAATCATCTTTCACAC
Peg3 outside	TGATAATAGTAGTTTGATTGGTAGGG	TAATTCACACCTAAAACCCTAAAACC
Peg3 inside	TTTTGTAGAGGATTTTGATAAGGAGG	AAATACCACTTTAAATCCCTATCACC
Snrpn outside	TATGTAATATGATATAGTTTAGAAATTAG	AATAAACCCAAATCTAAAATATTTTAATC
Snrpn inside	AATTTGTGTGATGTTTGTAATTATTTGG	ATAAAATACACTTTCACTACTAAAATCC
**ChIP Primers**		
Lmnb2	ACTCCCATAATTTATCCTCTGATGTC	GACAGGCTAAGTAAAGGATGTCTATTTTC
Snai3	ATTGCCGTCCCAGAGAAGGAT	TACACAGATATGGCCATTTGCC
Xist exon 7	GCCATCCTCCCTACCTCAGAA	CCTGACATTGTTTTCCCCCTAA
Fgf4	CAGCCCAAGATGGAAGAAGC	TGAGCCACCAGACAGAAAGG
Nanog	ATCAGAGGATGCCCCCTAAG	GAATTCACAGTTAATCCCACCTG
Oct4 DE (Figure S1A)	GGAACTGGGTGTGGGGAGGTTGTA	AGCAGATTAAGGAAGGGCTAGGACGAGAG
Lefty1	GCCTGCTTTCCAATCTCAAG	AGACTCGTCCCTGGTGTGTT
Xist intron 1	AACCCTTTTAAGTCCACTGTAAATTCC	TAGAGAGCCAGACAATGCTAAGCC
Tsix	TTAATGTTCAGTTTTCAATTCGGAAGAGAAG	GTTTGTCTGCCTACTAACACAGGTAAG
Xite	CAAGGTTGGGAACAAGGTATATCAGG	GGACAAGGGACAGAAGTGCTTATTTTAC
Oct4 DE (Figure 6B)	GCTGGGCTGCAGGCATACTT	AACCTCCCCACACCCAGTTC
Nr5a2	TTTTTCCACCCCTCCCACTT	GAACCCACCCAGTAGGCAAA
HoxB1	CTCCAACAAAACTCGGGGTA	ACTGAGAAGGCCCATAGCTG
Dusp16	CATCCGCGCTTGAAGTTT	AGGGTAAATGGTTGGAAACG
Nfat5	CAAAGTGCCCTGTTACAGCA	GGCCTACAAGCAATTCAAGC

SUPPLEMENTAL REFERENCES

Huang da, W., Sherman, B.T., and Lempicki, R.A. (2009). Systematic and integrative analysis of large gene lists using DAVID bioinformatics resources. Nat Protoc *4*, 44-57.

Irizarry, R.A., Hobbs, B., Collin, F., Beazer-Barclay, Y.D., Antonellis, K.J., Scherf, U., and Speed, T.P. (2003). Exploration, normalization, and summaries of high density oligonucleotide array probe level data. Biostatistics *4*, 249-264.

Kurimoto, K., Yabuta, Y., Ohinata, Y., and Saitou, M. (2007). Global single-cell cDNA amplification to provide a template for representative high-density oligonucleotide microarray analysis. Nat Protoc *2*, 739-752.

Langmead, B., Trapnell, C., Pop, M., and Salzberg, S.L. (2009). Ultrafast and memory-efficient alignment of short DNA sequences to the human genome. Genome Biol *10*, R25.

Lee, T.I., Johnstone, S.E., and Young, R.A. (2006). Chromatin immunoprecipitation and microarray-based analysis of protein location. Nat Protoc *1*, 729-748.

Lucifero, D., Mertineit, C., Clarke, H.J., Bestor, T.H., and Trasler, J.M. (2002). Methylation dynamics of imprinted genes in mouse germ cells. Genomics *79*, 530-538.

Ohinata, Y., Ohta, H., Shigeta, M., Yamanaka, K., Wakayama, T., and Saitou, M. (2009). A signaling principle for the specification of the germ cell lineage in mice. Cell *137*, 571-584.

Storey, J.D., and Tibshirani, R. (2003). Statistical significance for genomewide studies. Proc Natl Acad Sci U S A *100*, 9440-9445.

Tang, F., Barbacioru, C., Nordman, E., Li, B., Xu, N., Bashkirov, V.I., Lao, K., and Surani, M.A. (2010). RNA-Seq analysis to capture the transcriptome landscape of a single cell. Nat Protoc *5*, 516-535.

Touboul, T., Hannan, N.R., Corbineau, S., Martinez, A., Martinet, C., Branchereau, S., Mainot, S., Strick-Marchand, H., Pedersen, R., Di Santo, J.*, et al.* (2010). Generation of functional hepatocytes from human embryonic stem cells under chemically defined conditions that recapitulate liver development. Hepatology *51*, 1754-1765.

Ying, Q.L., Stavridis, M., Griffiths, D., Li, M., and Smith, A. (2003). Conversion of embryonic stem cells into neuroectodermal precursors in adherent monoculture. Nat Biotechnol *21*, 183-186.

Zhang, Y., Liu, T., Meyer, C.A., Eeckhoute, J., Johnson, D.S., Bernstein, B.E., Nusbaum, C., Myers, R.M., Brown, M., Li, W.*, et al.* (2008). Model-based analysis of ChIP-Seq (MACS). Genome Biol *9*, R137.
